# Prospects of Nanotechnology in Clinical Immunodiagnostics

**DOI:** 10.3390/s100706535

**Published:** 2010-07-07

**Authors:** Anees A. Ansari, Mansour Alhoshan, Mohamad S. Alsalhi, Abdullah S. Aldwayyan

**Affiliations:** 1 King Abdullah Institute for Nanotechnology, King Saud University, Riyadh-11451, P.O. Box-2455, Saudi Arabia; E-Mails: malsalhy@gmail.com (M.S.A.); dwayyan@hotmail.com (A.S.A.); 2 Department of Chemical Engineering, College of Engineering, King Saud University, Riyadh-11451, P.O. Box-2454, Saudi Arabia, E-Mail: alhoshanm@gmail.com; 3 Department of Physics and Astronomy, College of Science, King Saud University, Riyadh-11451, P.O. Box-2455, Saudi Arabia

**Keywords:** nanotechnology, antibody, antigen, nanomaterials, immune-biosensors

## Abstract

Nanostructured materials are promising compounds that offer new opportunities as sensing platforms for the detection of biomolecules. Having micrometer-scale length and nanometer-scale diameters, nanomaterials can be manipulated with current nanofabrication methods, as well as self-assembly techniques, to fabricate nanoscale bio-sensing devices. Nanostructured materials possess extraordinary physical, mechanical, electrical, thermal and multifunctional properties. Such unique properties advocate their use as biomimetic membranes to immobilize and modify biomolecules on the surface of nanoparticles. Alignment, uniform dispersion, selective growth and diameter control are general parameters which play critical roles in the successful integration of nanostructures for the fabrication of bioelectronic sensing devices. In this review, we focus on different types and aspects of nanomaterials, including their synthesis, properties, conjugation with biomolecules and their application in the construction of immunosensing devices. Some key results from each cited article are summarized by relating the concept and mechanism behind each sensor, experimental conditions and the behavior of the sensor under different conditions, *etc.* The variety of nanomaterial-based bioelectronic devices exhibiting novel functions proves the unique properties of nanomaterials in such sensing devices, which will surely continue to expand in the future. Such nanomaterial based devices are expected to have a major impact in clinical immunodiagnostics, environmental monitoring, security surveillance and for ensuring food safety.

## 1. Introduction

### 1.1. Nanostructured Materials

Nanostructured materials are a new class of materials which provide one of the greatest potentials for improving the performance and extending their applications in various fields of material sciences and technology, as well as biomedical sciences [1–25]. The study of nanostructured materials involves manipulation, creation and use of materials, devices and systems, typically with dimensions smaller than 100 nm. Nanostructured materials or matrices within this range display unique physical and chemical features because of effects such as the quantum size effect, mini size effect, surface effect and macro-quantum tunnel effect. Nanometer-scale materials display dominant physical properties which are different from those of their bulk counterparts. Therefore, these are key features of nanomaterials which play an important role in the advancement of nanotechnology in human healthcare [1,2,13,16–28]. Such progress in nanobiotechnology demands methods to observe, characterize and control phenomena at the nanometer-scale. In this article, we highlight the applications of nanostructured materials in the development of clinical immunodiagnostic devices.

#### 1.1.1. Nanostructured Conducting Polymers

Since the last decade nanostructured conducting polymers have played an important role in the development of nanobiotechnology [8,21,29–31]. Nanostructured conducting polymers exhibit tunable porosity, high surface area, low energy optical transitions, low ionization potential, high electron affinity and remarkable unusual electrical conducting properties [31–36]. A number of conjugated polymers have been transformed from an insulating into a highly conductive state. The large number of organic compounds which effectively transport charge can roughly be divided into three groups: *i.e.*, charge transfer complexes/ion radical salts, organometallic species and conjugated organic polymers [33,34]. Moreover, polymers are relatively inexpensive and can be functionalized using various patterning methods to achieve required optical, electronic or mechanical properties, and they also demonstrate biocompatibility. These unique features of nanostructured materials have led to a variety of applications in analytical sciences, biosensor devices and drug release systems, as reviewed by various researchers [31–35]. New materials have been fabricated and the possibilities of surface modification of conventional electrodes have been expanded, providing new and interesting properties which can be used in the development of biosensing devices. Numerous articles describe electrochemical nanobiosensors based on polymeric nanomaterials [29–33]. Recently, some investigators have discussed the fundamental nature and interpenetration of polymeric nanomaterials in biosensing and immunosensor technology [33,34].

#### 1.1.2. Metal Nanoparticles

The utilization in analytical chemistry of novel metal nanoparticles as nanoscale optical biosensors and immunosensors is beginning to receive significant attention. Due to their unique optical and electrocatalytic properties, nanoparticles of some noble metals (e.g., platinum, palladium, gold, silver) and carbon nanotubes (single walled and multi-walled) have been employed to design and develop modern biosensors [3–14,37]. Noble metal nanoparticles often display extraordinary electrocatalytic activity in many reactions like CO oxidation, catalytic hydrogenation of unsaturated alcohols and aldehydes and O_2_ reduction, compared to the corresponding bulk metal species that show lesser or even fairly poor electrocatalytic activity in the same reactions [8,14]. The catalytic performance of the metal nanoparticle-based electrodes was found to depend markedly on the particle size, the nature of the support as well as the method of preparation of the nanoparticles [14,37,38].

#### 1.1.3. Nanostructured Metal Oxides

Nanostructured metal oxides are known for their high mechanical, chemical, physical, thermal, electrical, optical, magnetic and also specific surface area properties, which in turn define them as nanostructures, nanoelectronics, nanophotonics, nanobiomaterials, nanobioactivators, nanobiolabels, *etc.* [4,7,39]. In the last decade a large variety of nanostructured metal oxide (ZnO, TiO_2_, ZrO_2_, SnO_2_, CeO_2_, MnO_2_, Fe_3_O_4_ and SiO_2_) devices with new capabilities have been generated [40]. The semiconducting, piezoelectric and pyroelectric properties of these ceramic nanostructured metal oxides find interesting applications in optics, optoelectronics, catalysis, as sensors and actuators and in piezoelectricity. Nanostructured metal oxides have wide band gaps and higher binding energy (ZnO = 60 meV) and are optically transparent and reflective, thus making them ideal candidates for fabrication of ultraviolet light-emitting diodes and lasers. Moreover, nanostructured metal oxides not only possess high surface area, nontoxicity, good bio-compatibility, high isoelectric point (IEP) and chemical stability, but also show biomimetic and high electron communication features, giving them great potential in biosensor manufacturing. A number of reports have been published in the literature on the use of nanostructured metal oxides for the construction of immunosensors [40].

#### 1.1.4. Semiconductor Nanoparticles or Quantum Dots

Colloidal semiconductor nanoparticles, also termed “quantum dots” (QDs) are 10^−9^-meter scale nanocrystals, smaller than their exciton Bohr radii, that are neither small molecules nor bulk solids [41–45]. These materials have several extraordinary optical and spectroscopic properties, including size-dependent tunable photo-excitation and emission with narrow and symmetric luminescence spectra. Their size-dependent optical and electronic properties can be tuned by changing the particle size, which is controlled by altering their synthesis procedures. The principle behind this unique property is the quantum confinement effect. This leads to differently sized QDs emitting light of different wavelengths that becomes shorter as their size decreases. On absorbing light, semiconductor QDs quickly re-emit the light but in a different color with longer wavelength, which is fluorescent. These size dependent fluorescent semiconductor nanocrystals interact with biological systems at the molecular level and facilitate the detection of multiple biomolecules. QDs have led to a new era in nanotechnology for the optical detection of pathogens [41,45].

#### 1.1.5. Nanostructured Organic-Inorganic Hybrid Nanocomposites

Inorganic-organic hybrid nanocomposites have drawn the attention of many scientists over the last few years [46–50] because of their potential of combining the distinct physical properties of their organic and inorganic components within a single molecular composite. In particular, inorganic nanoparticles/conducting polymer nanocomposites combine the magnetic, optical, electrical or catalytic characteristics of the inorganic metal nanoparticles and the electrical properties of the polymers, which could greatly widen their applicability in the field of catalysis, electronics and optics [47–50]. Organic materials offer structural flexibility, convenient processing, tunable electronic properties, photoconductivity, efficient luminescence and potential for semiconducting and even metallibehavior [49,50], whereas inorganic metals provide the potential for high carrier mobilities, the band gap tenability, a range of magnetic and dielectric properties, thermal and mechanical stability. These heterogeneous nanocomposites can exhibit quite different characteristics than the individual materials, for example, electrical conductivity and stability of the ZrO_2_/polypyrrole nanocomposites is much improved compared to that of plain polypyrrole [51]. Their high electron communication features, large surface area, optical transparency and enhanced binding energies at organic-inorganic interfaces have been exploited in analytical sciences for biosensor devices.

### 1.2. Preparation of Nanomaterials

Recently, a variety of methods have been employed for the synthesis and growth of nanostructured materials [37,38,40,52–54]. Three commonly adapted strategies are physical vapor deposition (PVD), chemical vapor deposition (CVD) and solution-based chemistry (SBC) [52,53]. The physical vapor phase growth involves mainly the vapor-liquid-solid (VLS) growth as used in the synthesis of Ge nanowires. Oxide-assisted growth such as the silicon oxide-assisted growth of silicon nanowires by thermal evaporation and laser ablation also belong to the vapor phase method category. Chemical vapor deposition (CVD) for the synthesis of structure-dependent nanomaterials for multiple applications in sensing devices has been reported [39,40]. Furthermore, electrochemical and electrophoretic deposition methods have been applied for the fabrication of polymeric nanocomposites, inorganic-organic hybrid nanocomposites and 1D metal and metal oxide nanomaterials. Solution based chemistry like as the sol-gel, micro-emulsion, hydrothermal/solvothermal, sonochemical, co-precipitation and template assisted processes have also been widely used to prepare metal, metal oxide and semiconductor nanomaterials [52–54]. All these synthesis techniques, summarized in Table 1, have been discussed previously in detail [40,52].

#### 1.2.1. Physical Vapor Deposition (PVD)

**Figure 1.**
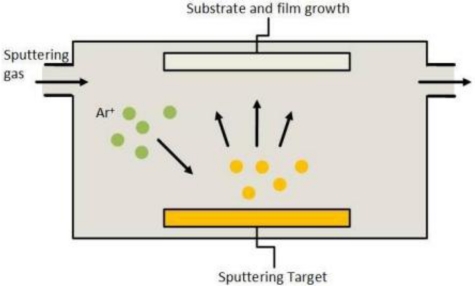
A schematic of the physical sputtering deposition technique.

Physical vapor deposition (PVD) is a process in which the vapor is created in a physical manner. The three most important techniques used for deposition of metal oxide films on glass substrates are sputtering, pulsed laser deposition, and thermal evaporation (Figure 1). These methods share in common the fact that the source material is the same as the intended depositing material and no chemical reactions occur throughout the process.

#### 1.2.2. Chemical Vapor Deposition (CVD)

Chemical vapor deposition (CVD) is a process used for the synthesis of nanomaterials in which one or more volatile precursors chemically react and/or decompose on the substrate surface to produce the desired material deposit. CVD processes differ from PVD in that a chemical reaction is necessary in creating the desired stoichiometry in CVD, whereas in PVD the desired stoichiometry is similar to that of the source material. Frequently, volatile byproducts of the chemical reaction are produced. Among the more common CVD techniques used to deposit ZnS are thermal CVD, molecular beam epitaxy (MBE), and atomic layer deposition (ALD). In each of these techniques, a vacuum chamber with a gas flow is required.

#### 1.2.3. Solution Based Chemistry (SBC)

**Figure 2.**
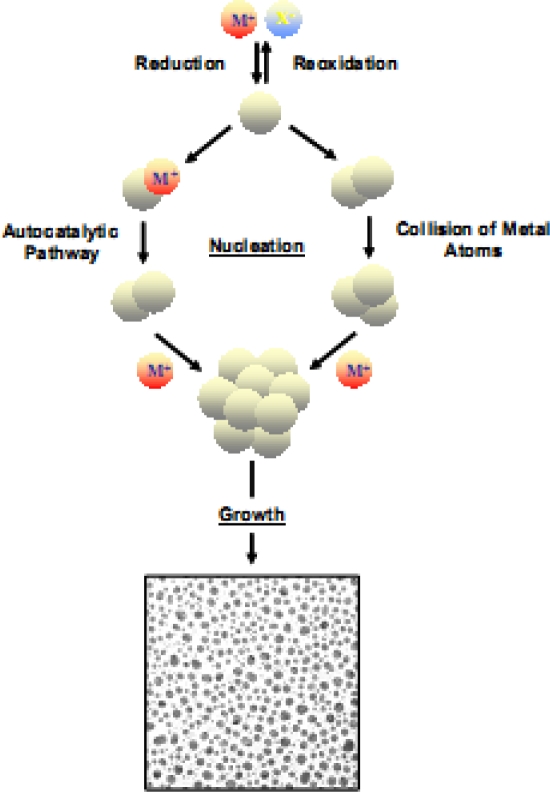
Formation of nanostructured metal colloids via the salt reduction method.

Any chemical reaction that requires use of a solution is a form of solution based chemistry (SBC). Some materials with complex stoichiometries are often difficult to synthesize via vapor deposition techniques. In these situations, SBC has served as a vital technique in producing these materials (Figure 2). SBC techniques typically provide materials with high yield and uniformity, but a major disadvantage is the increased point, line and planar defects compared with vapor deposition created materials. The most important technique for ZnO synthesis is the sol-gel process.

The so-called sol-gel chemical process is a type of solution based chemistry. Sol-gel techniques create inorganic networks through the formation of a colloidal suspension in a liquid (sol) and subsequent gelation of the solution to form a network in a continuous liquid phase (gel). Precursors for creating these colloids are metals/metalloids surrounded by various reactive ligands. Functionally, three reactions describe the sol-gel process: hydrolysis, alcohol condensation, and water condensation. The sol-gel process allows the fabrication of materials with a large variety of properties: ultra-fine powders, monolithic ceramics and glasses, ceramic fibers, inorganic membranes, thin film coatings and aerogels. The sol-gel process has been used to fabricate metal oxide films for different purposes such as photoelectrodes, gas sensors, biosensors and also phosphor applications.

#### 1.2.4. Electrochemical Deposition Methods

In the electrochemical method, an electrolyte consisting of metal ions and the reductant is used, along with two inert electrodes. When an appropriate potential is applied between the electrodes, the reductant is oxidized at the anode to yield electrons for the reduction. These electrons reduce the metal ions at the cathode to form metal nanoparticles. Surfactants added along with the electrolyte stabilize the as-formed nanoparticles. In addition, the simple isolation and high purity of the nanoparticles lie in the control of particle size (1–10 nm) achieved by adjusting the current density. Tetraalkylammonium salts are commonly used as surfactants and they serve simultaneously as supporting electrolyte and stabilizer for the nanoparticles. Martin and co-workers have demonstrated the formation of nanorods of gold and silver in the pores of a membrane by electrochemical methods. Nanoparticles prepared by this way could be isolated from the membrane by dissolving it in a suitable solvent [40,52].

#### 1.2.5. Physical Preparation Methods

High-energy milling and mechanical alloying have been studied by several researchers for the synthesis of nanocrystalline materials [52]. The basic concept behind this technique is the reduction of the grain size in coarse-grained powder samples to a few nanometers by heavy mechanical deformation followed by powder compaction. Initially the deformation is localized in shear bands with a thickness of about 1 μm. These shear bands act as nucleation sites for nanometer-sized grains. However, with increasing milling times, an extremely fine-grain microstructure in the nanometer range with randomly oriented grains separated by high-angle grain boundaries is produced.

### 1.3. Nanostructured Materials Properties and Applications

Recently the use of nanomaterials in biotechnology has been widely discussed due to the fact that nanomaterials effectively merge the fields of material science and biological sciences [52]. The interdisciplinary boundary between materials science and biology has become a fertile ground for new scientific and technological developments. For the fabrication of an efficient bioelectronic device, the selection of substrate for dispersing the sensing material decides the sensor performance. Micrometer scale materials usually exhibit physical properties similar to those of the bulk form; however, materials in the nanometer scale may exhibit distinctively different physical properties [37,40,52–54], and some remarkable alterations in physical, chemical and biological properties have been observed due to the increased surface area, biocompatibility, smaller particle size, reduced number of free electrons and quantum confinement effects [52,53]. In this section, we will discuss the main characteristics of nanomaterials, which play a very significant role in the fabrication of efficient bioelectronic sensing devices.

*Particle size*: Size effects have a dramatic impact on the structural, thermodynamic, electronic, spectroscopic, electromagnetic and chemical properties of nanomaterials such as CdS and CdSe. For example, semiconductor nanocrystals are zero-dimensional quantum dots, in which the spatial distribution of the excited electron-hole pairs are confined within a small volume, resulting in enhanced non-linear optical properties. Due to the reduction of particle size, the electronic properties of nanomaterials are significantly affected by the single electron transport mechanism, offering the possibility to produce single electron devices. These nanometer-scale electronic transducers reduce the pathway for direct electron communication between redox biomolecule to the electrode for sensitive and speedy detection of analytes without any hindrance [4,5,7].

*Surface chemistry*: Due to the large specific surface area of nanomaterials and their high surface free energy, nanoparticles can adsorb and covalently bind to the surface of biomolecules to impart high stability and rich linking chemistry to provide the desired charge and solubility properties [4,7,16–22,24,25]. The large surface-to-volume ratio of nanomaterials can change the role of surface atoms. The surface potential of nanomaterials plays an important role in the performance and characteristics of all devices involving surface chemistry such as semiconductor-based biosensors. Designer particles, including colloidal gold or inorganic nanocrystals, enhance the surface energy of the surface atoms for tagging biological macromolecules. The reduced coordination number of the surface atoms greatly increases the surface energy so that atom diffusion occurs at relatively lower temperature. In the case of gold nanoparticles, the melting temperature of gold nanoparticles drops to as low as ∼300 °C for particles with diameter smaller than 5 nm, much lower than the bulk melting point 1,063 °C for Au [14]. Fundamental studies of the surface potential have been vital to understand the behavior of these materials as well as their applications in chemical sensors and biosensors.

*Biocompatibility*: Biocompatibility of the nanomaterials is another important factor in the development of competent biosensing devices. After adsorption onto the surfaces of nanoparticles, the biomolecules can retain their bioactivity because of the biocompatibility of nanoparticles. Since most of the nanoparticles carry charges, they can electrostatically adsorb biomolecules with opposite charges. Besides the common electrostatic interaction, some nanoparticles can also immobilize biomolecules by other interactions. For example, it is reported that gold nanoparticles can immobilize proteins through the covalent bonding between the gold atoms and the amine groups and cysteine residues of proteins [7,8,14].

*Catalytic properties*: In general noble metal nanoparticles have excellent electrocatalytic properties [7,8,14–17]. Metal nanoparticles have been used as catalysts in innumerable biosensor applications, due to their superior stability and complete recovery in biochemical redox processes. The introduction of nanoparticles with catalytic properties into electrochemical sensors and biosensors can decrease the overpotentials of many analytically important electrochemical reactions without being self-consumed (*i.e.*, the catalyst may undergo several chemical transformations during the reaction, but, at the conclusion of the reaction, the catalyst is regenerated unchanged). For example, gold nanoparticles have excellent electrocatalytic behavior and bind tightly with the amido groups of suitably functionalized organic compounds through non-covalent electrostatic adsorption and can also form a powerful Au-S covalent bond with -SH groups [7,8,14–20]. In this way, the nanogold can integrate with the biological active components and the probe formed in this way can be used in the detection of a biological system. Platinum nanoparticles are another type of nanoparticle that exhibit good catalytic properties and have been used in the fabrication of electrochemical biosensing devices [7].

*Electrical conductivity*: Some nanomaterials exhibit remarkable electron transport properties, which are strongly dependent on their nanocrystalline structure. In particular, one-dimensional nanomaterial (carbon nanotubes, titania nanotubes, silica nanowires, polymeric nanowires and nanofibers) are the most attractive materials due to their different electrical conductance, which can be monitored by the change in electrical conductance of the fabricated electrode [4,5,10–13,52–54]. Electron transport properties of such nanomaterials are very important for electrical and electronic applications, as well as for understanding the unique one-dimensional carrier transport mechanism. It has been noticed that the wire length and diameter, wire surface condition, crystal structure and its quality, chemical composition, crystallographic orientation along the wire axis, *etc.*, are all important parameters which influence the electron transport mechanism of nanowires [4,5]. Because of the high surface-to-volume ratio and novel electron transport properties of these nanostructures, their electronic conductance is strongly influenced by minor surface perturbations (such as those associated with the binding of macromolecules). Such 1D materials thus offer the prospect of rapid (realtime) and sensitive label-free bioelectronic detection, and massive redundancy in nanosensor arrays. In particular carbon nanotubes are most exciting 1D nanomaterials that have generated considerable interest due to their unique structure-dependent electronic and mechanical properties.

The direct electron transfer ability of carbon nanotubes is another important factor that has been exploited in the fabrication of efficient electrochemical biosensing devices [3,8–12,]. For example, the use of single walled carbon nanotubes has enabled a direct electron transfer with the redox active centers of adsorbed oxidoreductase enzymes. Similarly, horseradish peroxidase adsorbed on a carbon nanotube microelectrode was found to transfer electrons directly to the electrode and retain its catalytic activity for H_2_O_2_ [48,54]. Carbon nanotubes enhanced the performance of bio-electronic devices partly due to the high enzyme loading and partly because of the better electrical communication ability of the nanotubes.

These distinctive properties and utility of such one-dimensional nanomaterials arise from a variety of attributes, including the similar size of nanoparticles and biomolecules such as proteins, enzymes, antibodies and polynucleic acids. Such bio-electronic devices based on one-dimensional nanomaterials are increasingly a potentially leading approach. One-dimensional nanomaterials make an efficient electronic interface whose sizes are comparable to the size of biological molecules. All these properties can be used as the basis for the development of analytically useful bioelectronic sensing devices, including chemical sensors, biosensors, and bio-chem-FETs. This is due to the fact that external chemical stimuli can drastically alter these fundamental and easily measurable surface properties of the nanomaterials.

## 2. Biosensors (Immunosensor) as Diagnostic Tools

Biosensors (immunosensors) can be defined as quantitative or semi-quantitative detection analytical techniques (devices) containing a sensing biomolecule (antibody) which can convert a biological signal into an electrochemical or optical signal [45]. An immunosensor is a sensitive interface including a bioreceptor coupled with a transducer able to detect binding events between the bioreceptor (antibody) and the analyte (antigen). Immunosensors provide a rapid and convenient alternative to conventional analytical methods for detecting and in some cases measuring an analyte in a complex medium. Classically different classes of biosensors are distinguished, and among them the immunosensor is a type of biosensor that exploits the ability of an antibody to recognize its associated antigen in a very complex medium [1,2,26,27]. The principle of immunoassays was first established by Yalow and Berson in 1959 [55], and later on in 1962 Clark and Lyons [56] developed the concept of immunosensor. Nowadays, immunosensors are applied widely in many fields such as clinical chemistry, food industry and environmental analysis.

The original methodology involved immobilizing an antibody on the surface of the electrochemical sensors so as to use the selectivity of the antibody for analytical purposes. Such a specific molecular recognition of antigens by antibodies has been exploited in immunosensors to develop, for example, highly selective detection of proteins. Antibody-antigen interactions are by their very nature complexations and it follows that the affinity reaction must be only minimally perturbed by the fabrication procedure to allow the immunosensors to display reproducible response characteristics. These reproducible electrochemical or optical response signals are dependent on the binding characteristics between immobilized or mixed biomolecules (antibodies) on the transducer surface with the analyte of interest (antigens). The molecular antibody-antigen interaction is dependent on the force of attraction between the two specific molecules (antibody-antigen) that is strongly bound and forms an immune-complex.

The interaction of the biomaterial with an antibody is a fundamental feature for developing an immunosensing electrochemical device. Most of the immunosensing devices involve the formation of recognition complexes between the sensing biomaterials and the analyte (antibodies or antigens) on the thin film surface of the biomaterial to configure the electrochemical transducer [57–59]. The immobilized antibodies form a complexation on the surface of the fabricated nanostructured material electrode. Formation of the complexation on conductive or semiconductive nanostructured materials surface alters the current, capacitance and resistance properties of the solid support-electrolyte interface.

### 2.1. Transducers for Molecular Recognition

A transducer is a detector device, frequently employed in the quantitative analysis of an analyte in biological samples, that converts a biological response signal (change) resulting from the interaction with the target analyte into a quantifiable electrical signal. The biological sensing element responds to the analyte being measured and the transducer converts this observed change into a measurable signal that is proportional to the concentration of the analyte. The designed devices contain the appropriate offset and the amplification circuits that enable the small electrochemical or optical signal change due to the enzymatic reaction to be measured. The selectivity of the immunosensor for the target analyte is mainly determined by the bio-recognition element, whilst the sensitivity of the biosensor is greatly influenced by the transducer. A variety of traditional transducers are used, such as electrochemical, optical and mass sensitive (piezoelectric) ones.

#### 2.1.1. Electrochemical Transducer

The name electrochemical biosensor is applied to a molecular sensing device which intimately couples a biological recognition element to an electrode transducer. According to the IUPAC definition, electrochemical transducers are integrated devices, which are able to provide specific quantitative or semiquantitative analytical information using a biological recognition element (biochemical receptor) retained in direct and spatial contact with the transduction element. The major processes involved in electrochemical biosensor system are analyte recognition, signal transduction and readout.

**Figure 3.**
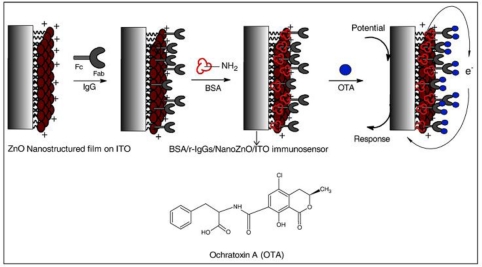
Schematic of the fabrication of a BSA/r-IgGs/Nano-ZnO/ITO immunosensor along with the biochemical reaction between ocratoxin-A (OTA) and immunosensor.

Due to their specificity, easy operation, quick response, portability, eco-friendly and cost effective, biosensors offer exciting opportunities for numerous decentralized clinical applications ranging from ‘alternative-site’ testing (e.g., physician’s office), emergency-room screening, bedside monitoring or home self testing. Therefore, electrochemical devices have traditionally received the major share of the attention in biosensor development. Such devices produce a simple, inexpensive, accurate and sensitive platform for patient diagnosis. The electrochemical transducers are classified based on the electronically amplified signal most commonly being utilized are amperomatry, impedomatric, potentiomatric and conductomatric (Figure 3).

##### Amperometric Transducers

Amperomatric transducers quantify the current of a redox species (antibody or enzyme) that is typically immobilized onto an amperomatric electrode at an applied potential, which is correlated to the concentration of analyte in solution. The amperomatric biosensor is fast, more sensitive, precise and accurate than the potentiometric ones discussed below, therefore it is not necessary to wait until thermodynamic equilibrium is obtained and the response is a linear function of the concentration of the analyte. However, the selectivity of the amperomatric devices is governed by the redox potential of all the electro-active species present, and consequently, the current measured by the instrument can include the contribution of several chemical species. There are four common ways that an amperomatric device is constructed: (1) an oxygen consuming antibody is immobilized onto a working electrode and platinum is used as a reference electrode, then the reduction of oxygen at the electrode produces a current that is inversely proportional to the analyte concentration; (2) an alternate approach is to provide for direct or mediated electron transfer from the electrode to the enzyme, thus eliminating the oxygen consumption at the electrode; (3) utilizing an biomolecules (enzyme or antibody) directly immobilized to a polarized anode to produce hydrogen peroxide; the detection limit for hydrogen peroxide based sensor is generally better than for oxygen sensing systems, but the selectivity is usually poorer; and (4) oxidizing the analyte with a dehydrogenase antibody.

##### Impedometric Transducers

The electrochemical impedometric (EI) transducer is the most commonly used transducer because of the combined analysis of both the resistive (conductance) and capacitive properties of the electrolytes it provides, based on the perturbation of a system at equilibrium by a small amplitude sinusoidal excitation signal [60,61]. An impedometric transducer can monitor the interface response of the electrode by applying a periodic small amplitude ac signal on the electrode. In this case, the adsorption or desorption of insulating materials on conductive supports can be assayed due to the change of the interfacial electron transfer features at the electrode surface. Compared with the other electrochemical methods mentioned above, EIS can provide information on the surface difference on the transducer before and after biological molecular interactions such as antigen–antibody and protein–protein. EIS immunosensors have been utilized for determination of DNA, protein, microorganism, drug small molecules and cell apoptosis *etc.* [62]. The potential of EI is that the impedance of the system can be scanned over a wide range of alternative current (AC) frequencies.

##### Potentiometric Transducers

Potentiometric transducer electrodes, capable of measuring surface potential alterations at near-zero current flow are the least common of all biosensors. When the cell current is zero the electrical potential difference between a working electrode and a reference electrode can measured by potentiometric transducers [63]. The reference electrode must provide a constant half-cell potential and the working electrode develops a variable potential depending on the activity or concentration of a specific analyte in solution. The change in potential is related to concentration in a logarithmic manner. The ion-selective electrode (ISE) for the measurement of electrolytes is a potentiometric transducer routinely used in analytical chemistry. The antibody catalyzed reaction consumes or generates a substance which is detected by the ion-selective electrode.

##### Conductometric Transducers

Conductimetric biosensors are based on the principle of change of conductivity of the medium when bio-molecules (enzymes or antibody) metabolize uncharged substrates, such as an antibody [64,65]. This measurable change can detect small changes in the conductivity of the medium between two electrodes. The amount of charged metabolites is directly proportional to the growth rate of the organism and is easily quantifiable. Many biological membrane receptors may be monitored by an ion conductometric device using interdigitated microelectrodes. Conductometric transducers are usually not specific and have a poor signal/noise ratio, and therefore have been little used.

#### 2.1.2. Optical Transducers

In the development of biosensor technology, optical transducers play a vital role in direct chemical and biochemical analysis of toxic species commonly found in human and environment [19,20,25]. Optical transducers are the most convenient and simplest methods for direct biomolecule detection. In them an optical event, such as a change in UV–Vis absorption, electrophotoluminescence, bio/chemiluminescence or fluorescence/phosphorescence color, is caused by the interaction of the biocatalyst with the target analyte [24,25,66–73]. Some optical transducers are based on several transduction modes like shifts of the refractive index, reflectance, surface plasmon resonance, interferometry and wave guide coupling detection schemes [74–78].

Initially, optical sensors were developed for oxygen, carbon dioxide and pH using acid-base indicators, but later they have been extended for the construction of fluorescent and luminescent optrodes. Optrodes are constructed with an immobilized selective biocomponent at one end of an optical fiber, with both the excitation and detection components located at the other end. The intensity of absorbed or emitted light from the indicator dye changes upon interaction with the selective biocomponent, as is the case that the pH, pO_2_ and pCO_2_ fiber-optic probes achieve transduction *via* the indicator dye alone [69]. This change is directly proportional to the amount of analyte present in the sample. The principle of these fiber-optic probes is the total internal reflection phenomenon in a light guide using evanescent waves. An electromagnetic wave that exists at the surface of many forms of optical waveguides is used to measure changes in refractive index at the sensor surface [52,79]. These transducers had extended the limits of application of the spectrophotometric methods in analytical chemistry, specially, for miniaturized systems.

#### 2.1.3. Mass Sensitive Transducer

Recently mass sensitive transducers have become more popular due to the fact that they provide label-free on-line analysis for antigen–antibody interactions and also provide the option of several immunoassay formats, which allow for increased detection sensitivity and specificity. Quartz crystal microbalance devices are mass sensitive detectors that operate on the basis of an oscillating crystal that resonates at a fundamental frequency [80–82]. After the crystal has been coated with a biological reagent (such as an antibody) and exposed to the particular antigen, a quantifiable change in the resonant frequency of the crystal occurs, which correlates to mass changes at the crystal surface. The vast majority of acoustic wave biosensors utilize piezoelectric materials as the signal transducers. Piezoelectric materials are ideal for use in this application due to their ability to generate and transmit acoustic waves in a frequency-dependent manner. The physical dimensions and properties of the piezoelectric material influence the optimal resonant frequency for the transmission of the acoustic wave [83].

## 3. Application of Nanostructured Materials to Immunosensors

Immunosensors are based on the special reactions between antibody and antigen, which have been successfully applied in many fields such as food industry, environmental monitoring, biotechnology, pharmaceutical chemistry and clinical diagnostics [84]. In the development of immunosensor devices the use of nanostructured materials represents a new trend that is expected to have a big impact on the future of nanoscience. Nanostructured materials provide new approaches for developing new materials with dimensions on the nanoscale based upon molecular self-assembly, leading to speculation as to whether we can directly control matter on the atomic scale. Because of the small grain size of these nanomaterials and consequently the large volume fraction of atoms in or near the grain boundaries, these nanostructured materials exhibit outstanding properties that are often superior and sometimes completely new in comparison with those of conventional coarse-grained materials. These outstanding properties include increased chemical and thermal stability, enhanced diffusivity, high electrical conductivity (CNT, Au, Pt and polymeric nanostructured materials), reduced electrical resistivity, piezoelectric, pyroelectric, nontoxicity, biocompatibility, and superior soft magnetic properties [40]. These nanomaterials have exceptional optical and electrical properties due to the electron and phonon confinement effects and are receiving a great deal of attention as alternative matrices for antibody immobilization to improve stability and sensitivity of immunosensors. Nanostructured materials provide high surface area for higher antibody loading and a biocompatible microenvironment, thus helping the antibody to retain its bioactivity. Besides this, they provide direct electron transfer between antibody active site and electrode. The high surface-to-volume ratios, catalysis or catalyst supports, nanoscale metallic, metal oxides, semiconductors and polymeric nanomaterials provides a noticeable changes in their electrical conductance upon surface modification, which is useful for fabricating special nanodevices. Some nanostructures such as nanotubes, nanofibers, nanorods, nanowires, nanobelts and nano-diskettes have been explored in the development of nanobiosensors [3–8]. These structure based nanomaterials provide improved performance for the construction of devices for the sensitive, selective and quantitative detection of analytes (antigens). The application of nanostructured materials in the construction of immunosensor devices enables one to substantially enhance the concentration sensitivity as well as the throughput of analytical measurement systems, while lowering their cost [85–87]. Essential changes in the physicochemical properties of substances on their conversion to the nanostructured state make it possible to create efficient targeted devices. Their uniqueness is partially due to the very large percentage of atoms at interfaces and partially due to quantum confinement effects. A large number of reviews and articles on the topic of immunosensors are available in the literature [1,7,8,14–16,26,27]. In the present review, we provide a brief overview of recent studies using nanostructured materials with metallic, metal oxide, semiconductor, and polymeric materials.

### 3.1. Electrochemical Immunosensors

Electrochemical immunosensors have been extensively studied because of their specific features like fast response, ease of fabrication, high sensitivity, selectivity and quantitative detection of target analyte [85–96]. The high sensitivity of such devices, coupled to their compatibility with modern nanofabrication technologies, portability, low cost (disposability), minimal power requirements and independence of sample turbidity or optical pathway make them excellent candidates for immunosensor development. In the development of electrochemical immunosensors, nanostructured materials are currently the most active research field because of their small grain size, high electrocatalytic activity, nontoxicity, biocompatibility, good selectivity, tremendous specific surface area, high mechanical strength, enhanced chemical and thermal stability and negligible swelling in aqueous and non-aqueous solutions make them ideal materials for the construction of electrochemical immunosensor devices [84–96]. Major reports in the field of electrochemical immunosensor design are now focusing on electrochemical immunosensors utilizing electrochemical methods, including nanostructured polymeric materials, colloidal gold, carbon nanotubes, platinum, palladium nanoparticles, semiconductor nanoparticles and nanosized metal oxides, which have been successfully applied to immobilize antibodies for direct antigen detection [86–106]. Their nanosized structures are capable of detecting small concentrations of antigens. The direct electron transfer between the antibody and modified electrode has been observed when such nanomaterials are used to modify the surface of working electrodes. Therefore, nanosized materials possess excellent electron transfer rates, which are much better than those of conventional material electrodes, and they also allow surface chemistry for tethering foreign biomaterials such as antibodies and nucleic acids.

In particular, electrochemical immunosensors have been extensively studied due to their high sensitivity and simple instrumentation. In most studies, antibodies or antigens can be immobilized through various approaches, which include the immobilization by physical adsorption, intercalation in polymer membranes or through intermediator membranes and self-assembled monolayers (SAMs) [96,97]. Due to the lack of electrochemical activity of the antibodies or antigens, however, most electrochemical immunoassay techniques rely on the labeling of either the antigen or antibody or the use of probe molecules, such as ferricyanide, in the solution, which may add complexity to the immunoassay system. Several reports on electrochemical immunosensors have been recently published in literature based on immobilization of mediator molecules onto the electrode surface [84–119].

#### 3.1.1. Amperomatric Immunosensors

Amperomatric immunosensors have the advantage of being highly sensitive, usually small, robust, rapid, inexpensive and easily used outside the laboratory environment. Amperomatric immunosensors are designed to measure a current flow generated by an electro-oxidation/reduction (redox electrochemical) reaction at constant voltage catalysed by their antibodies, or by their involvement in a bioaffinity reaction on the surface of the working electrode [86–94]. The potential of the working electrode is maintained with respect to a reference electrode, usually Ag/AgCl, which is at equilibrium. Generally, most common working electrodes, e.g., noble metals like as platinum (Pt), gold (Au), graphite or modified forms of carbon, mixed oxides such as indium-tin-oxide (ITO), are being applied for the construction of amperomatric biosensors. Compared to other biosensing tools, amperomatric systems usually show linear concentration dependence over a defined range. Amperomatric immunosensors are well suited for antigen detection at lower concentration ranges. Amperomatric immunosensors can suffer from poor selectivity, especially when applied for oxygen/hydrogen peroxide detection, but this can usually be overcome by using mediators (e.g., ferrocyanide).

Generally metallic nanoparticles have been employed for the fabrication of amperomatric immunosensors [5,7,8,14,86–100]. Metallic nanoparticles can catalyze biochemical reactions and this capability is usefully employed in immunosensor design. Catalysis is the most important and widely used chemical application of metal nanoparticles and as such, has been studied extensively. Transition metals, especially noble metals (Au, Ag and Pt), show very high catalytic abilities for many organic reactions [8,14]. This catalytic behavior of the metallic nanoparticles is useful to enhance the amount of immobilized biomolecules in the construction of an immunosensor. Because of their ultrahigh surface area, colloidal Au nanoparticles are used to enhance the immobilization of IgGs on gold electrodes to ultimately lower the detection limit of the fabricated amperomatric immunosensor. Self-assembly of approximately 20 nm colloidal Au nanoparticles onto a thiol-containing sol-gel network modified gold electrode has been used for the construction of amperomatric immunosensor. Quantitative results for the designed (BGE)/MPS/Au/HBsAb electrode show a linear range for HBsAg of 2–360 ng mL^−1^, with a correlation coefficient of 0.998. The stability of the BGE/MPS/Au/HBsAb electrode when the immunosensor was stored in a dry state at 4 °C was investigated over a 30-day period. In the absence of gold nanoparticles the HBsAb electrode only retained approximately 30% of its initial sensitivity in the response to 120 ng mL^−1^ of HBsAg after 30 days. This immunosensor was applied to detect HBsAg in human serum samples [86]. The unique (AuNP–Ab_1_)(Ag)(Ab_2_–HRP) sandwich-like layer structure formed onto a gold electrode by self-assembly provides a favorable microenvironment to retain the bioactivity of the immobilized antobody and to prevent antibody molecule leakage. The excellent electrocatalytic activity toward H_2_O_2_ of the fabricated sandwich electrode indicated that the gold nanoparticles with HRP protein enhanced the electrocatalytic properties of the fabricated electrode for sensitive determination of antigen. The analyte detection limit of this immunoelectrode is 2 ng mL^−1^ or 2 pg μL^−1^ [120]. A nano-Au monolayer assembled onto a Nafion/thionine composite film modified on a gold electrode was applied for immobilization of α-1-fetoprotein antibody (anti-AFP). Immobilization of Thionine was attributed to the electrostatic force between positively charged Thi and the negatively charged sulfonic acid groups in Nafion polymer, whereas immobilization of nano-Au particles was attributed to the chemisorption of the amine groups of the Thionine and the opposite-charged adsorption. This factor enhanced the performance of the resulting immunosensor, such as the immobilization of antibody and high detection limit. This immunosensor displays a linear reponse in the 5.0 to 200.0 ng mL^−1^ concentration range with a detection limit 2.4 ng mL^−1^ that decreased after incubation at 37 °C for 10 min. This immunosensor exhibits good accuracy, high sensitivity, selectivity and long-term stability up to 120 days. The proposed method is economical and efficient, making it potentially attractive for clinical immunoassays [87]. Gold nanowires (Au NWs) and ZnO nanorod (ZnO NRs) composite film modified onto a glassy carbon electrode have been employed for rapid determination of α-1-fetoprotein in human serum samples. Scanning electron micrographs have been used for investigation of Au nanowires. Amperomatric response of the resulting immunosensor was in the 0.5–160.0 ng mL^−1^ range with a 0.1 ng mL^−1^ detection limit and stability was 4 months [88]. Colloidal gold nanoparticles with an average grain size of 13 nm were assembled onto a 3,3,5,5-tetramethylbenzidine/Nafion film-modified glassy carbon electrode, providing active sites for immobilization of antibody (anti-MIgG) molecules for rapid detection of antigen molecules (MIgG as a model analyte). The resulting immunosensor showed a linear amperomatric response in the concentration range of 4–120 ng mL^−1^ and the detection limit was estimated to be 1.0 ng mL^−1^. This immunosensor retained 90% of the initial current response after 30 days [89]. In another strategy, self-assembled gold nanoparticle monolayers were developed on the working electrode for amperomatric detection of α-1-fetoprotein in human serum samples. The linearity of the proposed immunosensor was varied in the concentration range 15–350 ng mL^−1^ with a detection limit of 5 ng mL^−1^ and the stability was found be up to 90 days [90]. Ou *et al.* used layer-by-layer assembly of gold nanoparticles, multi-walled carbon nanotubes-thionine (MWCNTs-THI) and chitosan on 3-mercaptopropanesulfonic acid sodium salt (MPS)-modified onto a gold electrode surface for amperomatric detection of carcinoembryonic antigen. The linearity was examined in two concentration ranges, 0.5–15.0 ng mL^−1^ and 15.0–200.0 ng mL^−1^, and the detection limit for carcinoembryonic antigen was found to be 0.01 ng mL^−1^. This immunosensor showed no change on storage at 4 °C for up to three months [91]. The combination MWCNTs and chitosan with nano-gold on the surface of a GC electrode seems to be a very promising technique for detection of carcinoembryonic antibody (CEA). Chitosan polymer allows for the covalent immobilization of CEA antibody, while the interference-free antigen determination is achieved due to the electrocatalytic properties of CNTs and nano-gold. Under optimal conditions the fabricated immunosensor can detect CEA concentrations in two ranges of 0.3–2.5 and 2.5–20 ng mL^−1^, with a detection limit of 0.01ng mL^−1^ [121].

Song *et al.* fabricated a sensitive reagentless amperometric immunosensor for sensitive determination of CEA concentrations. A combination of chitosan and MWCNTs with gold nanoparticles formed a layer on the glassy carbon electrode. A Prussian blue nanoparticles (PBNPs) layer was introduced onto the fabricated electrode as a redox probe to increase the electrochemical behavior of the bioelectrode. This nanocomposite electrode displayed high stability, biocompatibility, high electrochemical activity and efficient absorption of anti-CEA. The proposed immunosensing strategy offered a simple and convenient methodology for sensitive detection of CEA concentration ranges from 0.3 to 120 ng mL^−1^ with a detection limit of 0.1 ng mL^−1^ [122]. This same group has applied another approach for the amperometrical detection of α-1-fetoprotein based on a gold colloidal nanoparticles (GNPs) doped chitosan (CS)–iron oxide nanocomposite (CS–Fe_3_O_4_–GNPs). Horseradish peroxidase enzyme was used to block the possible active sites and Prussian blue used as a redox probe for electrochemical signal amplification. This immunosensor has a linear response in the 0.05 to 300 ng mL^−1^ concentration range with a detection limit of 0.02 ng mL^−1^ [123]. Cui and Zhu *et al.* used a strategy for determination of human IgG from real samples, using gold nanoparticles dispersed into colloidal carbon sphere applied onto a glassy carbon electrode. This gold nanoparticles and carbon spheres hybrid material improved the electrocatalytic behavior of the immunoelectrode. Gold nanoparticles improve the enzymatic activity and stability of HRP-labeled immunoconjugate for the oxidation of o-phenylenediamine by H_2_O_2_. The proposed immunosensor provided linear response over the concentration range between 5–250 ng mL^−1^ with a detection limit of 1.8 ng mL^−1^ [124].

Another interesting approach was described by Shi *et al.* [92], whereby bilayer films of gold nanoparticles and TiO_2_ nanoparticles were assembled onto a gold electrode for immobilization of CEA. The amperomatric response showed that the reduction current of the immunosensor decreased linearly in two CEA concentration ranges, 0.3–10 ng mL^−1^ and 10–80 ng mL^−1^, with a detection limit of 0.2 ng mL^−1^, in presence of 0.7 mM H_2_O_2_ as an analyte solution. This immunoelectrode exhibited stability up to 10 days [92]. The amperomatric performance of the self-assembled titania nanoparticles/gold nanoparticles composite electrode shows an excellent electrocatalytic activity toward the working electrode. This immunosensor exhibits excellent response performance to CEA under the linear range of 0.2–80.0 ng mL^−1^ with a detection limit of 0.07 ng mL^−1^ in the presence of 0.55 mM H_2_O_2_ in the working solution. Moreover, the good biocompatibility and large specific surface area of gold nanowires make them ideal for the adsorption of antibodies [93]. Furthermore, the immunosensor shows rapid response, high sensitivity, good reproducibility, long-term stability and freedom of interference from other coexisting electroactive species. A similar nanocomposite material-based CEA immunosensor was developed by modifying a gold electrode with gold nanoparticles and a SiO_2_/thionine nanocomposite self-assembled monolayer. An l-cysteine (Cys) layer was modified on a bare gold electrode for functionalization and uniform orientation. Different conditions were optimized on the electrode including adsorption time of the first nano-Au layer, the pH of HAc–NaAc buffer, temperature and incubation time, and the proposed electrode was proven to be sensitive and specific to detect CEA between 1.00 and 100.00 ng mL^−1^ with a detection limit of 0.34 ng mL^−1^, which was well within the normal physiological range. The capability of the modified electrode was optimized for direct CEA quantification in human blood serum samples [94]. In another report, Lin *et al.* employed a colloidal gold nanoparticle-modified chitosan membrane on indium-tin-oxide electrode for CEA determination. SEM observation was used to verify the successful immobilization of antibody on the electrode surface. Under optimized conditions the proposed immunosensor shows a linear range for CEA concentrations from 2.0–20 ng mL^−1^ with a detection limit of 1.0 ng mL^−1^. The proposed electrode is cost effective, has good stability, is biocompatible, nontoxic and can be mass-produced industrially [95].

Yuan *et al.* applied a [Ag–Ag_2_O]/SiO_2_ nanocomposite material for the determination of carcinoembryonic antigen. To connect the Ag–Ag_2_O nanoparticles on the SiO_2_ surface, 3-aminopropyl-trimethoxysilane was used as a connector to absorb silicon oxide nanoparticles. The incorporation of 3-aminopropyltrimethoxysilane into the nanoparticles provided enhanced surface properties such as a large surface, excellent conductivity and good redox activity. The electrochemical behavior of the fabricated immunosensor was optimized by cyclic voltammetry. This immunosensor is specific towards CEA in the range of 0.5–160 ng mL^−1^ with a detection limit of 0.14 ng mL^−1^, which was well within the normal physiological range. The electrode lost only 5.0% of its initial response after about 90 continuous measurements and retained 92.3% of its initial response up to 10 days [125]. A unique sandwich-like layer [amino-group functionalized mesoporous silica nanoparticles-thionin-horseradish peroxidase-secondary anti-human IgG antibody (MSN–TH–HRP–Ab_2_)] was assembled on a glassy carbon electrode for immobilization of primary anti-human IgG antibody for determination of human IgG antigen concentration. High catalytic efficiency of the proposed immunosensor was measured due to the presence of HRP and thionin. This immunosensor exhibited high sensitivity, reproducibility and showed a linear response within the concentration range of 0.01–10 ng mL^−1^ human IgG [126].

For the amperomatric determination of hepatitis B surface antigen (HBsAg), Zhuo *et al.*, employed gold nanoparticles and a horseradish peroxidase (HRP) modified gold electrode. HRP was used instead of bovine serum albumin (BSA) as a blocking reagent. The linearity of the system was optimized for determination of HBsAg concentration in the concentration range of 2.56–563.2 ng mL^−1^ with a detection limit of 0.85 ng mL^−1^. No obvious change was found in the amperomatric response over 120 days [96]. Zhao *et al.* developed a disposable amperomatric immunosensor for detection of *Vibrio parahaemolyticus* (VP) based on a screen-printed electrode (SPE) coated with agarose/nano-Au membrane and horseradish peroxidase (HRP) labeled VP antibody (HRP-anti-VP) [97]. HRP is a positively charged species that blocks the active sites of gold nanoparticles. The screen printed electrode displayed an amperomatric response in the range of 10^5^–10^9^ CFU mL^−1^ with an associated detection limit of 7.374 × 10^4^ CFU mL^−1^ (S/N = 3). A nanosensor was designed using a polyvinyl-butyral sol-gel film doped gold nanowire modified gold electrode for construction of a testosterone amperomatric immunosensor. Analytical results suggest that the performance of the polyvinyl butyral sol-gel film doped with gold nanowires was improved due to the presence of the gold nanowires, which provide stability and negative charge that greatly amplify the amount of antibodies immobilized on the electrode surface and improve the sensitivity and detection limit of the immunoelectrode. This immunosensor was optimized for the testosterone concentration range from 1.2 to 83.5 ng mL^−1^ with a detection limit of 0.1 n gmL^−1^ (at 3δ) and the as-prepared immunosensor were used to analyze testosterone in human serum specimens [98].

Owino *et al.* utilized a polythionine (PTH)/gold nanoparticles (AuNP)-modified glassy carbon electrode (GCE) for immobilization of aflatoxin B1-bovine serum albumin (AFB1-BSA) [99]. HRP was used for the interface between the antigen and the modified electrode to improve the electrocatalytic behavior of the electrode. The experimental results shows the response decreases as the concentration of free aflatoxin1 increases in the dynamic range 0.6–2.4 ngmL^−1^ and the detection limit was 0.07 ng mL^−1^. The results indicate that this procedure eliminates the need for the enzyme-labeled secondary antibodies normally used in conventional ELISA-based immunosensors [99]. A homogeneous mixed solution of diphtheria antibodies and colloidal silica/gold/silver nanoparticles modified platinum electrode was employed for construction of immunosensor. Immobilization of antibodies on the electrode surface was confirmed by potentiometric, cyclic voltammetry and electrochemical impedance techniques. The resulting immunosensor exhibited fast potentiomatric response (<3 min), high sensitivity and good reproducibility. Sensitivity of up to 960 ng mL^−1^ was achieved for antigen detection with a detection limit of 0.8 ng mL^−1^. The constructed immunosensor reveals satisfactory results as given by ELISA method, an alternative approach for detecting diphtheria antigen in clinical diagnosis [100]. Nafion film containing gold nanoparticles modified onto a glassy carbon electrode was utilized for direct determination of paraxon antibodies. The resulting immunosensor is highly selective (−0.03 mV) and sensitive to monitoring the concentrations of paraxon antibodies in aqueous samples of up to 1,920 μg L^−1^, with a detection limit of 12 μg L^−1^ [101]. Amperomatric and potentiometric immunosensors based on gold nanoparticles/tris(2,2-bipyridyl)-cobalt(III) multilayer films were applied for hepatitis B surface antigen determination. A layer of plasma-polymerized Nafion film (PPF) was deposited on a platinum electrode surface, then positively charged tris(2,2-bipyridyl) cobalt(III) [Co(bpy)_3_^3+^] and negatively charged gold nanoparticles were assembled on the PPF-modified Pt electrode using the layer-by-layer technique and hepatitis B surface antibody (HBsAb) was electrostatically adsorbed on the gold nanoparticles surface. This immunosensor was used to analyze HBsAg in human serum samples and results were compared to those obtained from the standard ELISA method [102]. Fu *et al.* used a novel method for immobilization of antibody on sandwich type electrodes (BSA/antibody/AuNPs/Au electrode) for the amperometric determination of antigen. Polymeric bionanocomposites (polydopamine) with platinum nanoparticles exhibited high catalytic efficiency for antibody loading and sensitive detection of antigen because the polydopamine provides high biocompatibility, adsorbability, and processibility [127].

Some new materials for the fabrication of amperomatric immunosensors are of great interest in the pursuit of increased stability of electrode-bound proteins, improved orientation of the immobilized antibodies, and an increase in protein loading. Magnetic nanoparticles as special biomolecule-immobilizing carriers provide an alternative matrix for the construction of immunosensors. Owing to their attractive properties such as superparamagnetic behavior and high surface area, magnetic nanoparticles have been used in immunology and successful applications of magnetic nanoparticles in the immobilization of biomolecules have been reported [103–106]. The use of magnetic nanoparticles as immobilization materials offers the following advantages: (1) more specific surface area for the binding of larger amounts of biomolecules; (2) lower mass transfer resistance; (3) selective separation of the immobilized biomolecules from a reaction mixture on application of a magnetic field. Among these materials, Fe_3_O_4_ magnetic nanoparticles are the most commonly studied. Their good biocompatibility, strong superparamagnetism, low toxicity and easy preparation process make them an essential candidate for the fabrication of electrochemical biosensors. [103–107]. For instance, Li *et al.*, studied the covalent binding of goat-anti-IgG antibody (IgGAb) to SiO_2_-coated Fe_3_O_4_ magnetic nanoparticles, which were surface modified with amino groups [108].

Liu *et al.* developed a magnetic nanoparticle-based amperomatric immunosensor by using magnetic beads and gold nanoparticle labels. Anti-IgG antibody-modified magnetic beads were attached to a carbon paste transducer surface by a magnet that was fixed inside the sensor [103]. Gold nanoparticle labels were capsulated to the surface of magnetic beads by sandwich immunoassay. Highly sensitive electrochemical stripping analysis offered a simple and fast method to quantify the captured gold nanoparticle tracers. The stripping signal of gold nanoparticles was found to be proportionally related to the concentration of target IgG in the sample solution [103]. Three layer magnetic nanoparticles composed of Au–PB–Fe_3_O_4_ modified onto gold electrodes have applied for fabrication of an ultrasensitive and reproducible electrochemical immunosensor. A new signal amplification strategy was developed based on bienzyme functionalized electrode used for sensitive detection of CEA and α-fetoprotein (AFP) antigens. The experimental results show that the modified multi-labeled matrix exhibits satisfactory redox electrochemical and high enzyme catalytic activity for sensitive antibody detection. Comparative studies of bienzyme catalysis were undertaken to examine the amplification effect on the linear concentration range. In the presence of glucose enzyme, this immunosensor has linear response in the CEA concentration range of 0.01–80.0 ng mL^−1^ with a detection limit of 4.0 pg mL^−1^ and AFP from 0.014 to 142.0 ngmL^−1^ with detection limit of 7 pg mL^−1^, respectively, whereas in the absence of glucose linear ranges of 0.04–80.0 ng mL^−1^ for CEA and 0.074–142.0 ng mL^−1^ for AFP are obtained. This immunosensor could be regenerated by simply using an external magnetic field [85]. Another approach in the amperomatric immunosensor field concerns the assembly of a sensing platform based on the magnetic core/gold shell nanoparticles-functionalized biomimetic interface on multiporous polythionine modified magnetic carbon paste electrode (MCPE). The resulting magnetic nanocomposite brings new capabilities for electrochemical devices by combining the advantages of gold and core shell of Fe_3_O_4_ and provides an alternative way for higher loading of carcinoembryonic antibody (anti-CEA) on functionalized immunoelectrodes. Differential pulse voltammetric (DPV) measurements indicated the interaction of antibodies and showed a linear current response over the concentration range of 1.5–60 ng mL^−1^ with a detection limit of 0.3 ng mL^−1^ [103].

Recently, chitosan was widely used as an effective dispersant of Fe_3_O_4_ nanoparticles. An amperomatric immunosensor based on magnetic nanoparticle doped chitosan nanocomposite film modified GCE was used successfully to detect ferritin. It was found that chitosan enhanced the electroactive surface area of the immunoelectrode and accelerated the rate of electron transfer between the redox-active antibodies and the electrode. A low detection limit of 7.0 ng mL^−1^ ferritin was achieved with linearity over the concentration range of 20–500 ng mL^−1^. The proposed immunosensor has long term stability of up to three weeks, which suggests a promising alternative approach for detecting ferritin in clinical diagnosis [105]. In another interesting approach, chitosan was introduced into Fe_3_O_4_ nanoparticles to covalently immobilize rabbit immunoglobulin antibodies (IgGs) for ochratoxin-A detection. The differential pulse voltammetry results show linearity over the concentration range 0.5–6 ng dL^−1^ with low detection limit (0.5 ng dL^−1^) and a fast response time (18 s) [106]. Core–shell (CdFe_2_O_4_–SiO_2_) magnetic nanoparticles modified carbon paste electrode was employed for covalent immobilization of goat anti-human IgG antibody (anti-IgG) for the construction of an IgG immunosensor. BSA and HRP sandwich moieties served as blocking agent and redox mediators, respectively, for antibodies-antigen interactions, forming a polymeric network to enhance the immunosensing activities. The new strategy is suitable for monitoring sandwich immunoassay and antibody antigen interactions in connection with alkaline phosphatase tracers. Such amplified immunoelectronic assays allow the detection of IgGs up to 0.18 mg mL^−1^ and display linearity over a concentration range from 0.51–30.17 mg mL^−1^ [107].

Carbon nanotubes, a novel type of carbon material which possess many unique mechanical and electronic properties, high chemical stability and high surface-to-volume ratio, have been intensively researched for electrocatalytic and sensing applications [109–115]. There are two groups of carbon nanotubes: multi-walled carbon nanotubes and single-walled carbon nanotubes. Their biocompatibility and ability to facilitate electron transfer make them suitable candidates for immobilization of biomolecules [33]. This research was focused on their electrocatalytic behaviors toward the oxidation of biomolecules. Based on the high electrocatalytic activity of CNTs, these materials can be used as substrate for the immobilization of biological molecules while at the same time act as electrochemical signal transducers. For detection of prostate specific antigen (PSA) in human serum samples mouse monoclonal (5G6) to PSA antibodies were immobilized onto MWCNTs coupled to a GC electrode. The performance of the fabricated immunosensor showed sensitive determination of prostate specific antigen with a linear range from 0–60 μg L^−1^. The optimum immunosensor configuration has allowed a highly sensitive, fast and highly selective prostate specific antigen (PSA) from clinical samples [109]. In another approach amperomatric enzyme-linked immunoassays were developed based on a single walled carbon nanotube-modified pyrolytic graphite (PG) electrode for detection of human serum albumin. The immunoassay was carried out in sandwich design by using SWCNTs–HRP layers and casein/detergent was used to block non-specific binding sites. The detection limit of this immunosensor was determined to be 75 pmol mL^−1^ (75 nM). The observed experimental results show that the mediation of the immunosensors lowered the detection limit to 1 pmol mL^−1^ (1 nM), providing significantly better performance than alternative methods [110]. CNTs can act as both an electrode and an immobilization phase in an electrochemiluminescence (ECL)-based sensing device [111]. Poly(ethylene vinylacetate) (EVA) was used as binder to produce carbon nanotube–EVA–antibody composite sheets specific to α–fetoprotein. The immunoassay was carried out in sandwich design by exposing the CNT–EVA sheets to a sample containing α–fetoprotein (AFP) and anti-AFP antibodies conjugated with colloidal gold or Ru(bpy)_3_^2+^. The SEM observations and ECL measurements verified that sandwich immunoassay complexes were formed on the surface of the nanotube–EVA composites and that the formation of these complexes was biospecific. The ECL signal was linearly dependent on the concentration of AFP up to AFP concentrations of 30 nM and limit of detection was about 0.1 nM. An amperometric immunosensor was fabricated for sensitive detection of α–fetoprotein by modifying a layer of multiwalled carbon nanotube–silver nanoparticles (MWNT–Ag) nanocomposite and chitosan–MnO_2_ (CS–MnO_2_) onto glassy carbon electrode. A gold nanoparticles layer was electrodeposited on the electrode to immobilize anti-AFP. This system can optimize the AFP concentration in the range of 0.25–250 ng mL^−1^ with a detection limit of 0.08 ng mL^−1^ (S/N = 3) [128].

A carbon nanotube thick-film composite screen-printed immunosensor was constructed using polysulfone (PS) as binder [112]. This matrix retained the RIgG antibody at the surface of a screen-printed electrode. The combination of MWCNTs, polysulfone and antibodies resulted into a novel composite material consisting of an interconnected CNT–polymer network and possessing mechanical flexibility, high toughness and high porosity. SEM photographs proved the significant difference in porosity between MWCNTs/PS and graphite/PS nanocomposites. The amperomatric measurement show six times higher sensitivity for MWCNTs biocomposite compared with graphite biocomposites. The MWCNTs/PS biocomposite retains the electrochemical behavior of the MWCNTs electrodes, the biocompatibility of PS binder acts as an integration matrix for all elements needed for the production of a complex biocomposite. An amperomatric immunosensor based on the adsorption of antibodies onto perpendicularly oriented assemblies of single wall carbon nanotubes called SWCNT forests was developed [113]. The unmediated sandwich immunosensor achieved a detection limit of 75 nM using HRP labels. However, mediation dramatically lowered the detection limit to 1 nM. The authors concluded that the difference between mediated and unmediated assays is due to the fact that the average distance between HRP labels and nanotube ends is too large for efficient direct electron exchange, which can be overcome by electron mediation. An electrochemical immunosensor for cholera toxin was developed based on poly (3,4-ethylenedioxythiophene)-coated carbon nanotubes [111].

Multiplexing capabilities of quantum dots were also demonstrated in connection to immunoassay. An electrochemical immunoassay protocol for the simultaneous measurements of proteins, based on the use of different inorganic nanocrystal tracers were described [115]. The multi-protein electrical detection capability was coupled to the amplification featured of electrochemical stripping transduction (to yield fmol detection limits) and with an efficient magnetic separation (to minimize non-specific adsorption effects). The multi-analyte electrical sandwich immunoassay involved a dual binding event, based on antibodies linked to the nanocrystal tags and magnetic beads. A carbonate linkage was used for conjugating the hydroxyl-terminated nanocrystals with the secondary antibodies.

**Figure 4.**
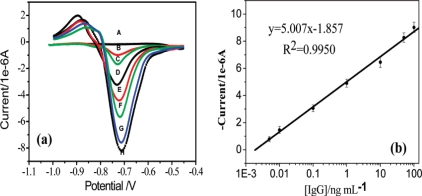
(a) Typical square wave voltammograms of electrochemical immunoassay with increasing HIgG concentration (from A to H: 0, 0.005, 0.01, 0.1, 1.0, 10, 50, and 100 ng mL^−1^ HIgG, respectively). (b) The resulting calibration curve of HIgG plotted on a semi log scale [116].

Each biorecognition event provided a distinct voltammetric peak, whose position and size reflected the identity and concentration of the corresponding antigen. The concept was demonstrated for a simultaneous immunoassay of *β_2_*-microglobulin, IgG, BSA and C-reactive protein in connection with ZnS, CdS, PbS and CuS colloidal crystals, respectively. In another approach, Liu *et al.* exploited quantum dots (QDs, CdS@ZnS) as labels for detection of prostate specific antigen (PSA) in human serum samples [115]. A similar strategy based on CdTe quantum dots was proposed by Cui *et al.* to immobilize antibody onto an Au nanoparticles monolayer modified on indium-tin-oxide glass plate for construction of an electrochemical immunosensor (Figure 4).

**Figure 5.**
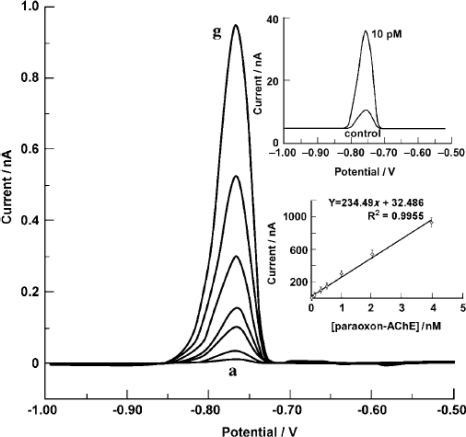
Typical electrochemical responses of the immunosensor with the increasing phosphorylated AChE concentration (0.01, 0.05, 0.3, 0.5, 1.0, 2.0, 4 nm from a to g). The insets show the resulting calibration plot (bottom) and the electrochemical responses of 10 pM and 0 pM (top) paraoxon-AChE. Each concentration was measured three times with three different immunosensors [118].

The resulting sandwich-type immunosensor provides a linear range 0.005–100 ng mL^−1^ by stripping voltametric analysis. The resulting immunosensor has good precision, high sensitivity, acceptable stability, reproducibility and could be used for the detection of real samples with consistent results in comparison with those obtained by the ELISA method [116]. The voltammetric response of the optimized device was linear over the concentration range from 0.1–10 ng mL^−1^ IgG, with a detection limit 30 pg mL^−1^ in association with 7 min immunoreaction times. The proposed disposable electrochemical diagnosis device provides a rapid, clinically accurate and quantitative tool for protein biomarker detection [117]. Potential applications of ZrO_2_/organophosphate and quantum dots (ZnS@CdS) as tags were proposed to monitor and quantify immune-recognition events (Figure 5). The electrochemical response of the immunosensor was highly linear over the range from 10 pm to 4 nm phosphorylated AChE, and detection limit was estimated to be 8.0 pm [118].

Efforts toward ultrasensitive electrochemical immunosensing (analogous to ultrasensitive DNA sensing) using carbon nanotubes loaded with enzyme were carried out with sensitivity down to 160 zmol of IgG and 1 amol for prostate specific antigen [114]. Kim *et al.* used an interesting approach to build an immunoelectrode based on AuNPs, dendrimers, CdS nanoparticles, [poly-5′,2′:5′,2″-terthiophene-3″-carboxylic acid, (poly-TTCA)] and the amino-derivative anti-chloramphenicol acetyl transferase (anti-CAT) antibody layer assembled onto GCE. To examine the morphological structure and electrocatalytic behavior, scanning electron microscopy (SEM), transmission electron microscopy (TEM), energy disruptive spectroscopy (EDS), X-ray photoelectron spectroscopy (XPS), quartz crystal microbalance (QCM) and cyclic voltammetry techniques were applied. This immunosensor has a CAP detection concentration range between 50 pg mL^−1^ and 950 pg mL^−1^ and a detection limit of 45 pg mL^−1^ and was also used to examine real meat samples for the analysis of CAP [129].

Yang *et al.* constructed a novel platform of Cu_2_O–SiO_2_ nanostructured particles modified gold electrodef for label-free amperomatrically detection of anti-ferretin antigen [119]. The chemical composition, porosity, surface morphology of the electrode and particles were confirmed using X-ray photoelectron spectroscopy (XPS), scanning electronmicroscope (SEM) and transmission electron microscopy (TEM) methods, respectively.

**Figure 6.**
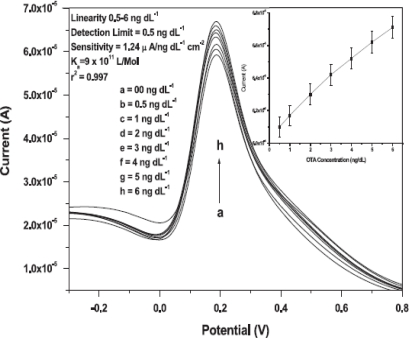
Electrochemical response studies of the BSA/r-IgGs/nanoCeO_2_/ITO immunoelectrode as a function of OTA using DPV, inset: calibration curve between the magnitude of current (A) and OTA concentration (ng dL^−1^) [130].

The proposed immunoelectrode had good electron transfer efficiency and large specific surface area with abundant electroactive sites. This immunosensor was sensitive and selective toward ferritin with linear concentration range covering from 1.0 to 5.0 and 5.0 to 120.0 ng mL^−1^ with a detection limit of 0.4 ng mL^−1^. The fabricated immunosensor could provide a low-cost, sensitive, quantitative detection of ferritin and could have potential application in clinical immunoassays with satisfactory results [119]. A novel sol-gel derived nanostructured cerium oxide (nanoCeO_2_) film deposited onto an indium–tin-oxide (ITO) glass plate was applied for immobilization of rabbit-immunoglobulin antibodies (r-IgGs) to quantitative detection of OTA (Figure 6).

The chemical composition and structural morphology of the modified electrodes before and after antibodies immobilization were confirmed by X-ray diffraction pattern and scanning electron micrographs. The proposed immunoelectrode have excellent electrochemical performance such as linear concentration range (0.5–6 ng dL^−1^), low detection limit (0.25 ng dL^−1^), fast response time (30 s) and high sensitivity (1.27 *μ*A ng^−1^ dL^−1^ cm^−2^) [130].

#### 3.1.2. Impedomatric Immunosensors

Electrochemical techniques are increasingly popular in the development of biosensor technology due to their rapid, accurate, selective and facile processing capability. Impedance spectroscopy is an electrochemical technique which is very powerful for the analysis of changes in the interfacial properties of modified electrodes upon biorecognition events that occur at modified surfaces. The concept of impedance, conductance, capacitance and resistance are only different ways of monitoring the test system and are all inter-related. The relationship between impedance (*Z*), resistance (*R*), capacitance (*C*), and frequency (*f*) for a resistor and a capacitor in series is expressed as follows: *Z*^2^−*R*^2−1^: (2*pfC*)^2^ [60–62]. Impedance is usually measured by a bridge circuit. Often a reference module is included to measure and exclude non-specific changes in the test module. The reference module serves as a control for temperature changes, evaporation, changes in amounts of dissolved gases and degradation of culture medium during incubation.

Impedance spectroscopy is an effective method for probing protein binding events such as antibody–antigen interactions. The formation of such bio-affinity complexes commonly leads to an insulating layer that retards the interfacial electron transfer kinetics between the redox probe and the electrode and increases the electron-transfer resistance. A small amplitude perturbing sinusoidal voltage signal is applied and the resulting current is measured. The impedance is the ratio between the voltage and the current [61]. Impedometrical detection of antibodies was found to be sensitive and rapid and it can detect at very low concentration, reaching a limit of detection of 10–18 mM in some cases. To directly investigate the binding reaction of antibody–antigen with electrodes, physical changes during the immune-complex formation are generally detected. Kim *et al.* applied an impedometric technique for *Salmonella enteritidis* detection, which detected impedance changes caused by the attachment of the cells to the anti-Salmonella antibodies immobilized on interdigitated gold electrodes [131]. The large surface area of the nanomaterials due to small grain size enhanced the loading of biomolecules (enzyme and protiens). The crystallinity and grain size of the nanostructured materials play crucial role in electrochemical activity of the fabricated nanomaterials based electrode, such as sensitivity, linearity, detection limit and reproducibility of the electrode. Covalent coupling of antibodies with functionalized gold nanoparticles were utilized for construction of electrochemical impedance immunosensor and probing apolipoprotein A-I. A hydrated indium-tin-oxide electrode was used for stepwise self-assembly of monolayers via the sol-gel technique. The average grain size of the gold nanoparticles was observed by TEM be 4 nm. UV/Vis and FTIR techniques were employed for structural characterization of the gold nanoparticles. The resulting modified electrode exhibited remarkable electrochemical characteristics such as higher protein loading, response time (6–17 times), reproducibility (R.S.D. = ±3.2%, *n* = 10), linear response to apolipoprotein A-I in the range of 0.1–10 ng mL^−1^. The detection limit of this immunosensor was found to be 50 pg mL^−1^ (corresponding to 1.8 pmol mL^−1^) [132]. Chen *et al.* developed a three dimensional ordered macroporous (3DOM) gold film modified electrode for the electrochemical impedomatric detection of C-reactive protein (CRP) [133]. The surface area of the fabricated 3DOM gold film modified immunooelectrode was measured by cyclic voltammetric (CV) and found to be 14.4 times higher than the classical electrode. CRP antibodies covalently conjugated with 3-mercaptopropionic acid (MPA) on the 3DOM gold film electrodes have excellent conductivity and stability. The dynamic linear concentrations of CRP were observed in the range of 0.1–20 ng mL^−1^ in the form of electron-transfer resistance (*R*et) values. This method is stable, versatile and highly sensitive and could be applied for other satisfactory immunoassays [133].

**Figure 7.**
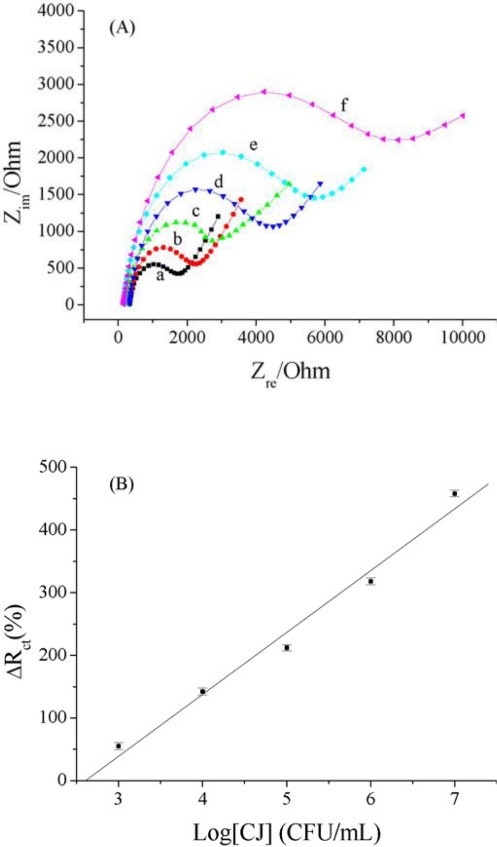
(A) Faradaic impedance spectra after the incubation of the immunosensor with different concentrations of CJ in 0.1M phosphate buffer solution (pH 7.0) and 0.1M KNO_3_ containing 1.0 mM Fe (CN)_6_^3−/4−:^ (a) blank solution, curves (b)–(f) represent 1.0 × 10^3^, 1.0 × 10^4^, 1.0 × 10^5^, 1.0 × 10^6^ and 1.0 × 10^7^ CFU mL^−1^ *Campylobacter jejuni*, respectively. (B) Calibration curve for the immunosensor [136].

A chitosan-containing titanium nanoparticles modified ITO immunoelectrode was constructed for immobilization of rabbit antibodies (r-IgGs) and proteins for sensitive detection of ochratoxin-A (OTA). Immobilization of antibodies and interaction of chitosan with titanium nanoparticles were investigated using by UV/Visible, FTIR spectroscopy. This immunosensor exhibited a linear response up to 10 ng mL^−1^ for detection of OTA [134]. Ansari *et al.* developed an impedomatric immunosensor based on sol-gel derived nanostructured ZnO film to immobilized rabbit antibodies (r-IgGs) for sensitive detection of ocratoxin-A [135]. Scanning electron microscopy and FTIR spectra were used to analyze the successful immobilization of r-IgGs on the fabricated electrode. The results of the impedometric immunosensor were linearity 0.25–6.0 ng dL^−1^ of mycotoxin with a detection limit of 0.25 ng dL^−1^, response time 15 s and sensitivity 0.46 Ωng dL^−1^ cm^−2^. Huang and coworkers reported the highly sensitive, fast response, long stability, wide linear range, and acceptable reproducibility of an electrochemical impedimetric immunosensor (Figure 7).

Impedometric methods applied for determination of *Campylobacter jejuni* concentration by immobilization of Anti-FlaA monoclonal antibodies 2D12 onto an O-carboxymethylchitosan surface modified Fe_3_O_4_ nanoparticles modified glassy carbon electrode. Such characteristics may be attributed to the electrocatalytic activity of O-carboxymethylchitosan surface modified Fe_3_O_4_ nanoparticles promoting the redox reaction between immobilized antibody and the surface electrode. Under optimized conditions the proposed immunosensor can detect *Campylobacter jejuni* concentrations in the range of 1.0 × 10^3^ to 1.0 × 10^7^ CFU mL^−1^ (r = 0.991) [136].

#### 3.1.3. Potentiometric immunosensors

Potentiometry is an old, well-established analytical instrumental method for sensing analysis [63]. Potentiometry is the measurement of an electrical potential difference when the cell current is zero between two electrodes, known as the indicator and reference electrodes. The reference electrode provides a constant half-cell potential and the indicator electrode develops a variable potential depending on the activity or concentration of a specific analyte in solution. The change in potential is related to the change in concentration in a logarithmic manner. The ion-selective electrode (ISE) for the measurement of electrolytes is a potentiometric technique routinely applied in immunosensors [137,138]. Furthermore, trace amounts of the analyte can be analyzed through this technique from the confined samples. There exists a direct relationship between sample activity and observed electromotive force, independent of the volume of the sample or electrode surface. Indeed, ion-selective microelectrodes were used for the detection of millimolar concentrations in single cells having volumes in the order of 1 pL. Thurer *et al.* applied a polymer membrane potentiometric sensor for ultra trace level detection of analyte (Cd^+^ ion) on the scale of nanomolar or lower concentrations [137]. The unique sandwich-like layer structure (microwell plat/IgGs/BSA/anti-mouseIgGs antigen/CdSe) was formed by self-assembly for monitoring the Cd^+^ ion, which is released by oxidation of CdSe nanoparticles dissolved in H_2_O_2_ solution. A Na^+^-selective electrode was utilized as pseudo-reference in order to facilitate measurements, which exhibited a log-linear response ranging from 0.15 to 4.0 pmol of IgG, with a detection limit of <10 fmol in 150 μL sample wells [137]. In another report, this group developed a disposable electrochemical immunosensor based on potentiometric stripping analysis of a metal tracer. For metal ion (Bi^+^) labeling human serum albumin (HSA) and anti-HSA antibody were used as a model system. This potentiometric immunosensor demonstrated a dynamic concentration range for HSA (0.3–30 μgmL^−1^) and good detection limit (0.2 μgmL^−1^, *i.e.*, 90 fmol in the 30 μL sample) [138].

#### 3.1.4. Conductometric immunosensors

Conductometric immunosensors measure the alteration of the electrical conductivity in a solution at constant voltage caused by biochemical (enzymatic) reactions, which specifically generate or consume ions from the immobilized biomolecules on the sensor surface. Most of the biomolecules (proteins and enzymes) produce ions that increase the electrical-conductivity of the electrode [63,65]. Conductometric immunosensors have attracted extensive attention with their high sensitivity, low cost, low power requirements and high compatibility with advanced micromachining technologies [64,65,139]. Furthermore, conductometric immunosensors are suitable for miniaturization and large-scale production, without reference electrode and with low driving voltage. The high sensitivity of the conductometric sensor makes it a good candidate for many other applications such as for sensor immuno-detection and few works report the use of conductivity for studying antigen–antibody interactions.

Recently, one-dimentional nanostructural materials (nanowires, nanotubes, nanofibers or nanobelts) were employed to greatly reduce immunosensor device dimensions [139]. These nanometer size materials possess larger surface area per unit mass and permit easier addition of surface functionalities compared with traditional materials which are highly agglomerated. The three-dimensional porous morphology of nanosize range materials allow easy electron communication into and out of the film and the nano-scale diameters lead to rapid electron transfer between the analyte (antibody) and electrode [65]. As a result, nanostructured-based conductometric immunosensor open a new avenue and platform in clinical conductometric immunoassays. The electrodes fabricated by electrochemical deposition of gold nanoparticles onto interdigitated microelectrodes (IDME) and immobilizing hepatitis B (HBs) antibodies conjugated with HRP for conductometrically detection of HBs antigen. SEM morphology confirmed the immunocomplex between the immobilized antibodies and nanogold. Analytical experimental results indicated the direct electrical communication between the carried HRP and electrode because local conductivity variations could be assayed based on the bioelectrocatalytic reaction of the carried HRP. Two linear concentration ranges were proposed for HRP-conjugated anti-HBs as secondry antibodies and double-codified nanogold particles from 1.5–450 ng mL^−1^ HBsAg and 0.1–600 ng mL^−1^ HBsAg, respectively. These optimized *anti*-HBs/protein/nanogold immunoelectrode shows significantly better performance, good precision, high sensitivity, acceptable stability and reproducibility [139]. Another approach in the conductometric immunosensor field concerns the assembling of a sensing platform based on magnetite nanoparticles for *Escherichia coli* (*E. coli*) detection. In this report, magnetite nanoparticles functionalized by biotin–streptavidin with cross-linking glutaraldehyde were used for immobilization of biotinylated antibodies anti-*E. coli*. Experimental results show excellent response as a function of antigen addition, the detection of 1CFU mL^−1^ *E. coli* induces a conductivity variation of 35 μS. The proposed conductometric immunosensor allows detection of 500 CFU mL^−1^ [65].

### 3.2. Optical Immunosensors

Undoubtedly, nanometer dimensional materials have demonstrated unique optical and electro-catalytic properties at room temperature. Therefore, these unique optical and electro-catalytic properties of nanomaterials were exploited for the fabrication of optical devices intended for clinical diagnosis such as optical biosensors and optical biolabeling or imaging [45]. Some luminescent nanomaterials (terbium and europium derivative nanoparticles), semiconductor quantum dots (QDs) and noble metal (gold and silver) nanoparticles are the most striking examples of unique optical properties arising from materials of nanometer dimensions, which are applied for construction of optical biosensors [65]. Optical-based biosensor systems are the most widely reported for construction of optical immunosensing devices [140–145]. Optical immunosensors can employ a number of techniques to detect the presence of a target analyte and are based on well-founded methods including chemiluminescence, fluorescence, phosphorescence, light absorbance, reflectance, light polarization and rotation. The modulation in optical properties such as UV–vis absorption, bio- and chemi-luminescence, reflectance and fluorescence brought by the interaction of the biocatalyst with the target analyte (antigen) is the basis for optical immunosensor. Optical based immunosensors offer advantages of compactness, flexibility, resistance to electrical noise and small probe size. Currently a number of techniques have been applied for the development of optical immunosensors [140–159].

#### 3.2.1. Fluorescence Immunosensors

**Figure 8.**
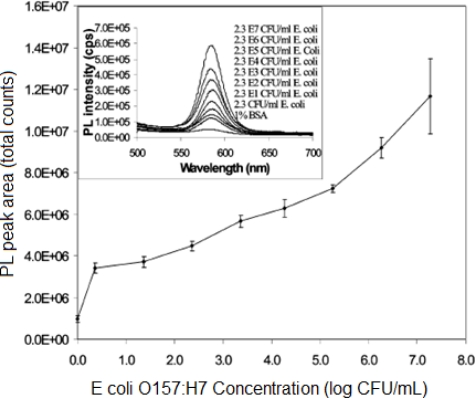
The curve of the photolu.minescence (PL) vs the concentration of *E. coli* O157:H7. Inset: PL spectra of the BSA and *E. coli* O157:H7 samples taken under the same conditions at the same excitation wavelength [143]

Fluorescence biosensors are an optical sensing technique applied in analytical chemistry for the detection of proteins, enzymes and antibodies due to their sensitive, selective and accurate detection of the biomolecules [45,140–145]. Fluorescence-based devices monitor the emission intensity of the nanomaterials, which is directly proportional to the concentration. In a typical fluorescence measurement, the fluorophore is excited by a specific wavelength of light and emit light at a different wavelength. Fluorescence sensors are capable to detect the analyte at very low concentration. In addition, nanofiberous membranes have also been introduced in the microfluidic immunoassays for HIV detection. The change in fluorescence signal (intensity) observed to determine the IgGs concentration [140]. Seydack *et al.* have discussed different kinds of nanostructured materials applied for optical detection of antigens [68]. The applicability of a variety of nanoparticles was summarized for the detection of antigen by optical-sensing technique. Inorganic nanoparticle labels based on noble metals, semiconductor quantum dots and nanoshells appear to be the most versatile systems for these bioanalytical applications of nanophotonics. Owing to the unique photophysical properties, quantum dots have been introduced as a model immunoassays system for detection of prostate specific antigen (TPSA) cancer marker from the spiked and undiluted serum samples. The resulting immunosensor shows an excellent response performance and high sensitivity, with a detection limit of 0.25 ng mL^−1^ [142]. Fluorescent CdTe@SiO_2_ nanoparticles were functionalized with avidin and biotin for sensitive detection of FITC-rabbit-anti-goat IgG antigens. The conjugation reaction of quantum dots, avidin and biotin with antibodies was confirmed by fluorescence spectra. The fluorescence intensity was altered by changing the antigen concentration in the solution. This immunosensor is applicable for the detection of FITC-rabbit-anti-goat IgG concentration upto 1.6 mg mL^−1^ [142]. Liu *et al.* illustrated surface modified CdSe/ZnS core/shell nanocrystals with the dendron ligands for detection of targeted pathogens, with *Escherichia coli* O157:H7 as an example for bacteria and hepatitis B [143]. The conjugation of antibodies with CdSe/ZnS core/shell nanocrystals sandwich structure complex (membrane-antigen-antibody conjugated with the nanocrystals) was measured through the photoluminescence spectra. The effect of pore size, buffer pH, detection limit (5 ngmL^−1^) and assay time (30 min) on the detection of *E. coli* were optimized (Figure 8).

However, they have not utilized the sensor for testing clinical samples [143]. The rabbit antihuman albumin IgG conjugated with EDCHCl, sulfo-NHS functionalized QDs (QD-carboxyterminated) have been utilized for efficient and sensitive immunoassays via the capillary electrophoresis technique [144]. Gold quantum dots (AuQDs) conjugated to goat-derived anti-human IgG demonstrated the specificity and a wide dynamic range for detection of human IgG. The proposed immunosensor shows linear fluorescence quenching over micromolar to nanomolar concentration range [145]. An average 20 nm grain size of gold nanoparticles conjugated with immunoglobulin G antibodies was employed for simple, ultrasensitive and fast immunoassay to biotin-peptide detection. Covalently attached biotin with antibody-coated gold nanoparticles exhibited improved linearity range from 1 pmol and 1 μmol to 100 zmol and 100 fmol for sensitive and quantitative immunoassay analysis. The linear working range of the immunoassay was increased between 100 zmol and 100 fmol by adding 100 zmol silver. Furthermore, this immunoassay can be extended to detect target molecules such as dioxin, digoxin, mercury, *etc.*, with appropriate matched antibodies [146].

**Figure 9.**
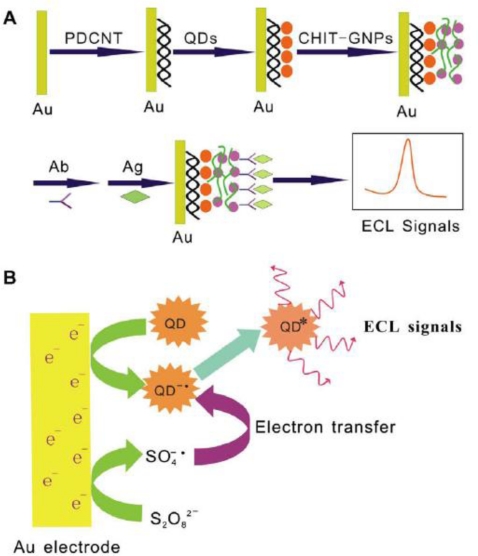
(A) The fabrication steps of the ECL immunosensor and (B) ECL mechanisms of the CdS QDs [151].

Zhang *et al.* presented a novel strategy based on optical absorption immunoassay for selective and sensitive detection of human serum albumin (HSA) antigen [147]. Antibodies (Ab) covalently immobilized onto a (3-aminopropyl) triethoxysilane-derivatized glass slide were utilized by cross-linking with glutaraldehyde (GA) based on combination of gold nanoparticles and chitosan as a label. The resulting immunosensor exhibited selectivity and sensitivity with a linear detection range of 8.0–512.0 μg mL^−1^. The presented biosensing method is simple and has low cost [147]. Gupta *et al.* used silver nanoparticles as sandwich immunoassays, which enhance the selectivity and sensitivity of the optical immunoassay [148]. In such a system, the optical signal enhancement was observed due to silver nucleation with gold nanoparticles for labeling antigens. They adequately optimized the fabricated sandwich immunoassay for high selectivity and reproducibility and found detection limits of antigen concentration up to 0.1 μg cm^−3^ (4 ng) [148]. Sharpe *et al.* introduced transmission spectra as an immunoassay based on cortisol thiol functionalized gold nanohole arrays for rapid detection of monoclonal antibodies [149]. The results of the optimized immunoassay indicate strong affinity, sensitivity and regenerable response. Jie *et al.* proposed a sensitive electro-chemiluminescence (ECL) immunosensor based on CdS quantum dots-carbon nanotubes (CNTs) and gold nanoparticles-chitosan for protein sensing in the clinical laboratory. The CdS QDs-CNTs exhibited highly intense ECL, good stability, which has great potential for specific, simple, fast and stable protein detection. This immunosensor was able to detect the human IgG antigen concentrations from 0.006 to 150 ng mL^−1^, and the detection limit was 0.001 ng mL^−1^ [150]. A similar strategy was reported by Tian *et al.* for ultrasensitive determination of antigen (human IgG) using an electrochemiluminescence (ECL) immunosensor based on luminol functionalized gold nanoparticles (Figure 9). This ECL immunosensor was able to detect HIgG concentrations in the range of 7.5–100 pg mL^−1^ with a detection limit of 1.0 pg mL^−1^. The proposed ECL immunosensor has been successfully applied to the detection of HIgG in real human serum samples [151].

#### 3.2.2. Surface Plasmon Resonance (SPR)

Surface plasmon resonance (SPR) imaging is a relatively new technique that combines the SPR method with spatially resolved measurement. Because of the high sensitivity of SPR to environmental changes, the SPR technique has received great attention, especially in biotechnological detection applications. Miniaturization, compactness, robustness, and multiassay sensing in one chip are among the most important characteristics of SPR biosensors configured with integrated-optical waveguides [74,75]. In comparison to other biosensing techniques, the biochemical detection based on SPR has advantages such as a high degree of sensitivity, no need for fluorescent labeling of the biomolecules before detection, real-time measurement of the kinetics of biomolecular interactions, and immunity from electromagnetic interference. SPR occurs as the surface plasma wave (SPW) is excited by the incident light wave. At that moment, the power of the incident light waves is transferred to the SPW such that its output power is obviously reduced. Typical SPR imaging sensors simultaneously monitor ten to hundreds of interactions that would more accurately characterize analytes in sample media. Several biosensor structures of this type have been proposed. In recent years, the applications of metal nanoparticles have received significant attention because of their unique properties, such as high surface-to-volume ratios and surface-enhanced Raman scattering. Krishnamoorthy *et al.* applied a self-assembled monolayer of nanoparticles on gold or silica substrates to monitor antibody-antigen interactions for the construction of a high efficiency one chip immunosensor [152]. X-ray photoelectron microscopy, scanning electron microscopy and atomic force microscopy techniques confirmed the interaction of the antibody-antigen with the fabricated SAMs layer of nanoparticles on gold plate. The optimized SPR immunosensor results indicated that antibody activity was increased 50% compared to non-patterned surfaces [152]. In another approach a layer of surface modified silica nanoparticles and gold capped nanoparticles was deposited onto self-assembled monolayer modified gold electrode (gold slide substrate) for detection of milk allergens casein. [153]. Atomic force microscopy (AFM) images demonstrated the formation of nanoparticle monolayers on the slide glass. Under optimized conditions, such a system could detect up to 10 ng mL^−1^ of casein.

#### 3.2.3. Surface-enhanced Raman scattering (SERS)

Surface-enhanced Raman scattering (SERS) based on vibrational spectroscopies has emerged as a powerful analytical tool that extends the possibilities of vibrational spectroscopy to solve a vast array of chemical and biochemical problems at the molecular level. SERS provides quantitative information for small molecule analytes based on their unique vibrational signatures [76]. It has inherent ability to distinguish between molecules with great similarity, such as structural isomers. SERS is not only useful for determining molecular structural information, but also provides ultrasensitive detection limits, including single molecule sensitivity and it has been used to detect pathogens, including bacteria and viruses [154–157]. Two primary mechanisms are believed to be responsible for SERS enhancement, which have been reviewed elsewhere [158–162]: a long-range classical electromagnetic (EM) effect [163] and a short-range chemical (CHEM) effect [164]. These two mechanisms contribute simultaneously to the overall enhancement; EM is thought to contribute the most (∼10^4^−10^7^) to the observed intensity enhancement, while CHEM is thought to contribute a lesser amount (∼10−10^2^).

SERS is currently utilized as an effective and powerful analytical tool in quantitative clinical biosensing applications [155]. However, one feature which has limited its use in such applications is the difficulty involved in producing uniform, highly sensitive and reproducible SERS substrates. Recent developments in oblique angle deposition and other nanofabrication techniques have overcome this limitation, providing an unprecedented opportunity to develop SERS substrates for pathogen biosensor applications. Functionalized SERS Au and Ag nanoparticles in the presence of Raman-active molecules are typically used in detecting, sensing, or imaging of biological samples such as DNA, proteins, antibody, cells, and tissues. The unique optical and electrical properties of Au and Ag nanoparticles make them useful tags for sensitive and selective measurement of antigens [155]. Due to their strong light absorption and light scattering capabilities, noble metal nanoparticles are useful for the quantitative detection of proteins, DNA and antibodies, as demonstrated by Mirkin’s research team [156]. Kim *et al.* prepared surface-enhanced Raman spectroscopic tagging material (SERS dots) composed of silver nanoparticle-embedded silica spheres and organic Raman labels for targeting cancer in living cells [157]. Silica shell encapsulated silver nanoparticles had excellent intense and consistent Raman signatures of the organic label compounds (4-MT, 2-NT, and TP) and maintained their own signatures with similar intensity after encapsulation by the silica shell. The resulting antibody conjugated SERS dots applied for targeting of HER2 and CD10 on cellular membranes [157]. In another approach, a gold nanoparticles surface was employed for measuring the SERS signal of a probe molecule (Crystal Violet, CV). The presented results indicate that the electrostatic interaction between thrombin and negative charged aptamer SAMs is weakened due to the electrostatic barrier effect [165]. The system showed highly specific selectivity and a linear detection of thrombin in the concentration range of 0.1−10 nM with a detection limit of 20 pM. Furthermore, the proposed immunoassay approach is promising for detection of thrombim in clinical samples. Nanostructured materials are promising in immunoassay development for the analysis of multiple biomolecules. Woo *et al.* used fluorescent surface-enhanced Raman spectroscopic dots (F-SERS dots) composed of silver nanoparticle-embedded silica nanospheres to detect biomolecules [166]. The results indicated that F-SERS dots are highly sensitive and selective for the detection of three cellular proteins, including CD34, Sca-1, and SP-C. Attempts have been made to use such immunoassays for the simultaneous analysis of multiple biomolecules as the primary next-generation labeling technologies.

### 3.3. Piezoelectric Immunosensors

Generally, piezoelectric materials are used for piezoelectric sensors. A piezoelectric device is portable, simple, cost effective and suitable for real-time monitoring of bio-specific interaction such as antigen–antibody with high sensitivity and specificity. Piezoelectric immunosensors are based upon the measurement of small changes in mass [80–83]. Piezoelectric sensors comprise a quartz crystal coated with a gold electrode and are used as microbalances (QCM) sensitive to changes in the mass on the sensor surface. The QCM technique has been used for a wide range of applications in the medical field to analyze biomolecule reactions such as antigen–antibody and DNA hybridization. QCM devices are extremely sensitive mass sensors, capable of measuring sub-microgram levels of mass changes [80]. The resonant frequency shift of a QCM is based on the measurement of mass change by the adsorption of target molecules. Piezoelectric immunosensors are usually designed to detect cancer markers where the specific antibody is immobilized on the sensor chip. More advanced devices are now available on the market based on this technology. Recently, microcantilever based sensors have also been applied for cancer biomarkers detection. Affinity interactions between the antibody on the surface of the cantilever and the biomarker are detected through the amount of bending of the sensor due to mass changes which are detected as changes in the resonant frequency [83]. Li *et al.* utilized amino-group functionalized magnetic nanoparticles to covalently immobilize antibodies ((IgGAb) for the construction of a piezoelectric immunoassay device [167]. SEM and TEM images confirm the covalent immobilization and surface modification of the immunoelectrodes. The process of immunoglobulin (IgGs) immobilization and immunoreactions with magnetic bio-nanoparticles attached to the surfaces of quartz crystal was monitored by frequency recording. The resulting piezoelectric immunosensor have ability to determine IgG in the range of 0.6–34.9 μg mL^−1^ with a detection limit of 0.36 μg mL^−1^ [167]. Goluch *et al.* constructed a single disposable chip based on functionalized gold nanoparticles for a bio-barcode assay (BCA) for screening single protein marker detection [168]. They tested the capability of the constructed immunosensor in the presence of prostate specific antigen (PSA) in buffer solution and goat serum. They found detection limit of PSA concentrations upto 500 aM. Krishnamoorthy *et al.* developed an effective protein immobilization technology with minimal amounts of protein (interleukin-6) for high sensitivity surface acoustic wave biosensors [169]. Furthermore, they determined the binding properties and morphological characteristics of human interleukin-6 (IL-6), a pro-inflammatory cytokine on the surface of ZnO, and SiO_2_ films grown onto (100) Si substrates. The immobilization of antibody and immunoreactions between antigen and antibody was monitored by change in relative mass value. They found that ELISA based immunoassays system can detect protein concentrations up to 1–6 ng mL^−1^ [170]. Carbon nanotubes can enhance the stability, specific surface area and fast electron communication features of the immobilized antibodies on aptamer-modified carbon nanotube field-effect transistors (CNT-FETs) for the detection of immunoglobulin E (IgE). Functionalized carbon nanotubes utilized as a gate material of field-effect transistor (FET) to covalent immobilization of 5′-amino-modified 45-mer aptamers on the CNT channels and electrical properties of the CNT-FETs were monitored in real time [170]. They found sharp decrease in the net source-drain current at various concentrations of target IgE and gradual saturation was observed at lower concentration. The performance of the antibody-modified CNT-FETs immunosensor indicate enhanced electrical properties after aptamer immobilization and low detection limit (250 pM). The fabricated aptamer-modified CNT-FETs are promising candidates for the development of label-free protein biosensors. Shen *et al.* developed a homogeneous self-assembled monolayer of cysteamine and 6-mercapto-1-haxanol onto AT-cut quartz crystal’s Au electrode surface to attach capped gold nanoparticles via glutaraldehyde for immobilization of carcinoembryonic antibody. Self-assembled monolayer increased the efficiency of antibody-antigen binding and minimized the nonspecific adsorption. Bovine-serum albumin was used to block the unnecessary possible remaining active sites for sensitive and specific detection of antigen. The resulting immunosensor have linear relationship of the CEA concentration within 25–500 ng mL^−1^ [171].

## 4. Conclusions and Future Prospects

This review has highlighted the applications of nanomaterials in the development of immunosensing devices. The emerging fields of nanotechnology and nanomaterials are opening new horizons in the construction of bio-electronic devices, since nanostructured materials represent a huge, well-established and important analytical field in clinical immunodiagnostics. Interest in the unique properties associated with materials having structures on a nanometer scale such as nanotubes, nanowires, nanorods, nanospheres, nanorings, nanoribbons, nanocomb, nanoflowers, nanofibers, nanoparticles, and nanocomposite materials had increasing number of applications in biomedical sciences. These nanostructured materials exhibits biocompatibility, non-toxicity, specific surface area, high chemical and thermal stability, electro-catalytic activity and fast electron communication features, which make them suitable materials as immobilization platform or labels for the sensitive recognition events. Structure-based nanoparticles not only provide stability to the sensing devices but also improve the sensing characteristics of the immunosensing devices, such as higher sensitivity, selectivity, linearity, fast response and reproducibility of the analytical devices.

In the success of electrochemical immunosensors, it has been observed that interaction of biological components with nanoelectronic plateform based on nanowires and nanotube structures is most promising and challenging. Nanowires are very robust materials based on their tensile strength, exhibit better sensing behavior, high electrical conductivity, good biocompatibility, and enhance the signal transduction between the nanoelectronic platform and the immobilized biomolecule for sensitive and fast detection of analyte. The unique properties of nanowires and nanotubes such as mechanical stability, light weight, enhancement in the current, reduction in potential are very helpful for the development of efficient electrochemical sensors and biosensors.

Therefore, comprehensive efforts should be made towards the synthesis of high-quality nanowires and nanotubes. A significant challenge still exist in their syntheses that include, but is not limited to, reliable control of diameter, length, orientation, density, crystallization and hierarchical assembly. The mechanism of interaction between biomolecules and nanomaterials is also not yet very well clarified. How to use these laws and principles of an optimized biosystem for fabricating novel multifunctional or homogenous nanofilms or modifying electrodes is also a great challenge. The processing, characterization, interface problems, availability of high quality nanomaterials, tailoring of nanomaterials, and the mechanisms governing the behavior of these nanoscale composites on the surface of electrodes are also great challenges for the presently existing techniques. For example, how to align nanomaterials such as CNTs in a polymer matrix along identical direction is a great challenge. How to enhance the signal to noise ratio and how to enhance transduction and amplification of the signals are also great challenges. Future work should concentrate on further clarifying the mechanism of interaction between nanomaterials and biomolecules on the surface of electrodes or nanofilms and using novel properties to fabricate a new generation of biosensors.

Sensitivity, selectivity and stability are the next generation electrochemical transductor parameters that will require significant improvements in order to meet the future demands in a variety of fields. Though some research groups have successfully detected electrochemical transduction signal of biological molecule using nanowires and nanotubes plateform, the selectivity and stability are still quite low.

We still hope to see continuing efforts to design and build more sophisticated circuits in which biological molecules work, interact, and communicate seamlessly with the nanotube and nanowire-based scaffolds, respond to electrical signal, or even perform fast reproducible electrochemical nanoelectronic devices.

Such nanostructure-based analytical tools are expected to have a major impact upon clinical diagnostics, environmental monitoring, security surveillance, and for ensuring food safety. However, this area is still on the horizon from the viewpoint of applied research. In order to fully exploit the potential application of nanomaterials in clinical chemistry for construction of immunosensing devices, more perfect nanoparticles with well-defined geometry, well-defined properties, and long-term stability in various environments have to be designed and synthesized.

## Figures and Tables

**Table 1.** Several deposition and synthesis methods of nanomaterials.

**SN.**	**General Techniques**	**Sub-techniques**
1.	Physical vapor deposition methods	Thermal evaporation Electron-beam RF induction Resistive *Sputtering* Focused ion beam Radio-frequency Magnetron sputtering Pulse laser deposition
2.	Chemical Vapor deposition (CVD) methods	Thermal CVD Low-pressor CVD Plasma-enhanced CVD Metal-organic CVD Molecular beam epitaxy (MBE) Atomic layer deposition
3.	Solution based Chemistry	Sol-gel chemical process Micro-emulsion method Sonochemical method Hydrothermal/solvothermal Co-precipitation Template-assisted synthesis
4.	Electrochemical synthesis	Electrochemical deposition Electrophoretic deposition
5.	Physical methods	High energy ball milling process
